# New virulence factor CSK29544_02616 as LpxA binding partner in *Cronobacter sakazakii*

**DOI:** 10.1038/s41598-018-19306-0

**Published:** 2018-01-16

**Authors:** Seongok Kim, Hyunjin Yoon, Sangryeol Ryu

**Affiliations:** 1 Department of Food and Animal Biotechnology, Department of Agricultural Biotechnology, Research Institute for Agriculture and Life Sciences, and Center for Food and Bioconvergence, Seoul National University, Seoul, 08826 Korea; 2 Department of Molecular Science and Technology, Department of Applied Chemistry and Biological Engineering, Ajou University, Suwon, 16499 South Korea

## Abstract

*Cronobacter sakazakii* is an opportunistic pathogen that can cause meningitis and necrotizing enterocolitis in premature infants, but its virulence determinants remain largely unknown. In this study, a transposon-mediated random-mutant library of *C. sakazakii* was used to identify new virulence factors. Compared to wild-type bacteria, a mutant lacking CSK29544_02616 (referred to as *labp*) was defective in invasion into intestinal epithelial cells (by at least 1000-fold) and showed less phagocytosis by macrophages (by at least 50-fold). The lack of *labp* in *C. sakazakii* changed the profile of outer membrane proteins, decreased the production of lipopolysaccharides, and increased the production of membrane phospholipids. Bacterial physiological characteristics including surface hydrophobicity and motility were also altered in the absence of *labp*, presumably because of changes in the bacterial-envelope structure. To systematically determine the role of *labp*, ligand fishing was conducted using Labp as a bait, which revealed LpxA as a binding partner of Labp. LpxA is UDP-*N*-acetylglucosamine (GlcNAc) acyltransferase, the first enzyme in the pathway of lipid A biosynthesis. Labp increased the enzymatic activity of LpxA without influencing *lpxA* expression. Considering multifaceted roles of lipopolysaccharides in virulence regulation, Labp is a novel virulence factor that promotes the production of lipid A by LpxA in *Cronobacter*.

## Introduction

*Cronobacter sakazakii* is an opportunistic food-borne pathogen that causes bacteremia, meningitis, and necrotizing enterocolitis, particularly in premature infants^1–3^. Despite its low incidence of infection, the mortality rate is high, ranging from 40% to 80%^1,4,5^. Recent studies revealed virulence determinants associated with *C. sakazakii* infection at the molecular level. For example, its flagellum functions as an immune stimulus for the production of pro-inflammatory cytokines within human-derived monocytes and outer membrane proteins including OmpA, OmpX, and Inv, which are important for bacterial invasion into host cells^6–8^. Lipopolysaccharides (LPS) are also essential for the invasion of *C. sakazakii* into intestinal epithelial cells via disrupting tight junctions^6^. Other virulence-associated genes in *C. sakazakii* include *zpx* encoding cell-bound zinc-containing metalloprotease, *cpa* encoding an outer membrane protease, *mcp* encoding a methyl-accepting chemotaxis protein, and *bcsABC* operon responsible for cellulose biosynthesis^9–12^.

The outer membrane of gram-negative bacteria is an asymmetric bilayer. The inner leaflet is enriched with diverse phospholipid (PL) compounds composed of glycerol, a phosphate group, and fatty acid moieties. The PL repertoire in the outer membrane varies between bacterial species. *Escherichia coli* and *Salmonella* Typhimurium possess phosphatidylethanolamine (PE), phosphatidylglycerol (PG), and cardiolipin (CL), which are the dominant PLs in the outer membrane, while others also contain phosphatidylcholine (PC) and phosphatidylinositol (PI)^13^. In contrast, the outer leaflet is abundant in LPS with a hydrophobic lipid A moiety anchored in the membrane, while its core oligosaccharide (inner and outer core) and O-antigen parts are exposed outward^14^. LPS are present at approximately 10^6^ copies per single *E. coli* cell, covering approximately 75% of the cell surface area^14,15^. Its heat-stable amphiphilic property protects bacteria from destructive agents, enabling them to maintain their structural integrity^16–18^. The lipid A is a highly conserved structure in gram-negative bacteria including *C. sakazakii* and consists of two glucosamine molecules that are generally coordinated with six hydrophobic acyl chains and two phosphate groups^19,20^. The core oligosaccharide attached to a glucosamine unit of lipid A is a short chain of sugars such as Kdo (3-deoxy-D-*manno*-oct-2-ulosonic acid), heptose, and hexose and is decorated with diverse substituents including phosphate, ethanolamine, and amino acids. This core structure is then connected to O-antigen, a hyper-variable polysaccharide region among bacterial species and different strains within the same species^20,21^. Of the LPS components (lipid A, core oligosaccharide, and O-antigen), the lipid A moiety is critical for bacterial viability. It is also responsible for the immune-activation abilities of LPS during bacterial infection^20^.

Several decades of investigations revealed that the lipid A biosynthesis pathway is complex, involving nine enzyme-catalyzed reactions^22^. UDP-GlcNAc acyltransferase encoded by *lpxA* participates in the first reaction in which the *R*-3-hydroxyacyl chain of *R*-3-hydroxyacyl-acyl carrier protein (ACP) is transferred to glucosamine 3-OH of UDP-GlcNAc, resulting in the formation of UDP-3-(*R*-3-hydroxyacyl)-GlcNAc^17,23–25^. A recent study showed that RipA, a cytoplasmic membrane protein conserved in *Francisella* species, can directly interact with LpxA and controls LpxA stability without affecting LPS production^26^. The following steps are orchestrated by numerous enzymes including LpxC, KdtA, LpxD, and LpxK. The activities of LpxC, UDP-3-(*R*-3-hydroxyacyl)-GlcNAc deacetylase, and KdtA, a Kdo transferase, are further regulated by the membrane protease FtsH, which degrades substrates of both enzymes^27,28^. Additionally, the catalytic activity of LpxK, a tetraacyldisaccharide 4′-kinase, is dependent on the concentration of PL, while the activity of LpxD, another *N-*acyltransferase, is modulated by NPr (encoded by *ptsO*) through a direct interaction^29^.

Because of the importance of lipid A in bacterial virulence and its conserved structure in gram-negative pathogens, lipid A biosynthesis enzymes are attractive targets for the development of new antibacterial agents^30^. For example, a pentadecapeptide known as peptide 920 directly bound to LpxA, resulting in bacterial growth inhibition^31^. In this context, peptide 920 has been exploited as a promising control agent against gram-negative pathogenic bacteria^20^.

The objective of this study was to identify new virulence factors important for virulence regulation in *C*. *sakazakii*. Labp encoded by CSK29544_02616 was found to interact with LpxA and eventually stimulated lipid A production. Therefore, *labp* is a promising target for designing novel strategies to control LPS production during *Cronobacter* infection, as *labp* is conserved among all *Cronobacter* species.

## Results

### CSK29544_02616 gene is important for *C. sakazakii* invasion into and survival inside mammalian cells

**Figure 1:**
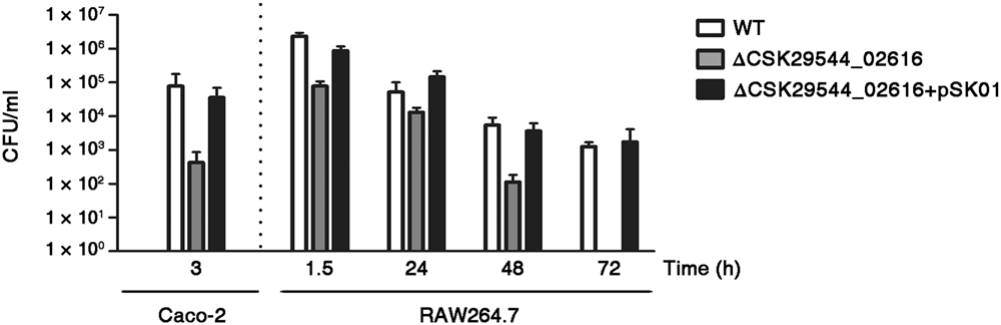
CSK29544_02616 is required for invasion into epithelial cells and survival within macrophages. Attenuation in invasion and survival because of the absence of CSK29544_02616. Confluent monolayers of Caco-2 epithelial cells or RAW264.7 macrophage cells were infected with three *C. sakazakii* strains: WT, ΔCSK29544_02616, and ΔCSK29544_02616 harboring pSK01. Plasmid pSK01 was designed to express CSK29544_02616 under its putative intrinsic promoter. Intracellular bacteria were enumerated at the indicated time points after infection.

To identify genes associated with the virulence of *C. sakazakii*, a transposon-mediated random-mutant library of *C*. *sakazakii* was constructed. Of 1200 clones, 300 were subjected to an invasion assay to identify clones defective in invasion into epithelial cells. The clone with the most attenuated invasion ability without growth defects contained a transposon inserted into the CSK29544_02616 gene (Supplementary Fig. S1). To validate the phenotype caused by disruption of CSK29544_02616, full-length sequences of CSK29544_02616 were deleted in-frame from the chromosome without an antibiotic-resistance marker. Compared to the wild-type (WT) strain, the deletion mutant lacking CSK29544_02616 was attenuated by at least 1000-fold in invasion to human-derived epithelial cells (Fig. 1). Complementation with pSK01, a plasmid harboring the CSK29544_02616 gene under its putative intrinsic promoter, fully restored the invasion ability of this mutant strain to the level of WT, thereby ruling out the possibility of polar effects or secondary site mutations in the constructed mutant strain. When bacterial persistence inside macrophage cells was evaluated using murine macrophage RAW264.7 cells, the ΔCSK29544_02616 strain showed 50-fold lower internalization by macrophages compared to the WT strain and was cleared out completely at 72 h post-infection, whereas WT bacteria persisted inside macrophages even at 96 h after phagocytosis (Fig. 1). Introduction of the pSK01 plasmid also successfully complemented the attenuated survival of this mutant strain inside macrophages (Fig. 1). These results indicate that CSK29544_02616 is required for intracellular persistence as well as invasion into host cells in *C. sakazakii*.

### Mutant lacking CSK29544_02616 is significantly attenuated in competition with WT *C. sakazakii* during host infection

**Figure 2:**
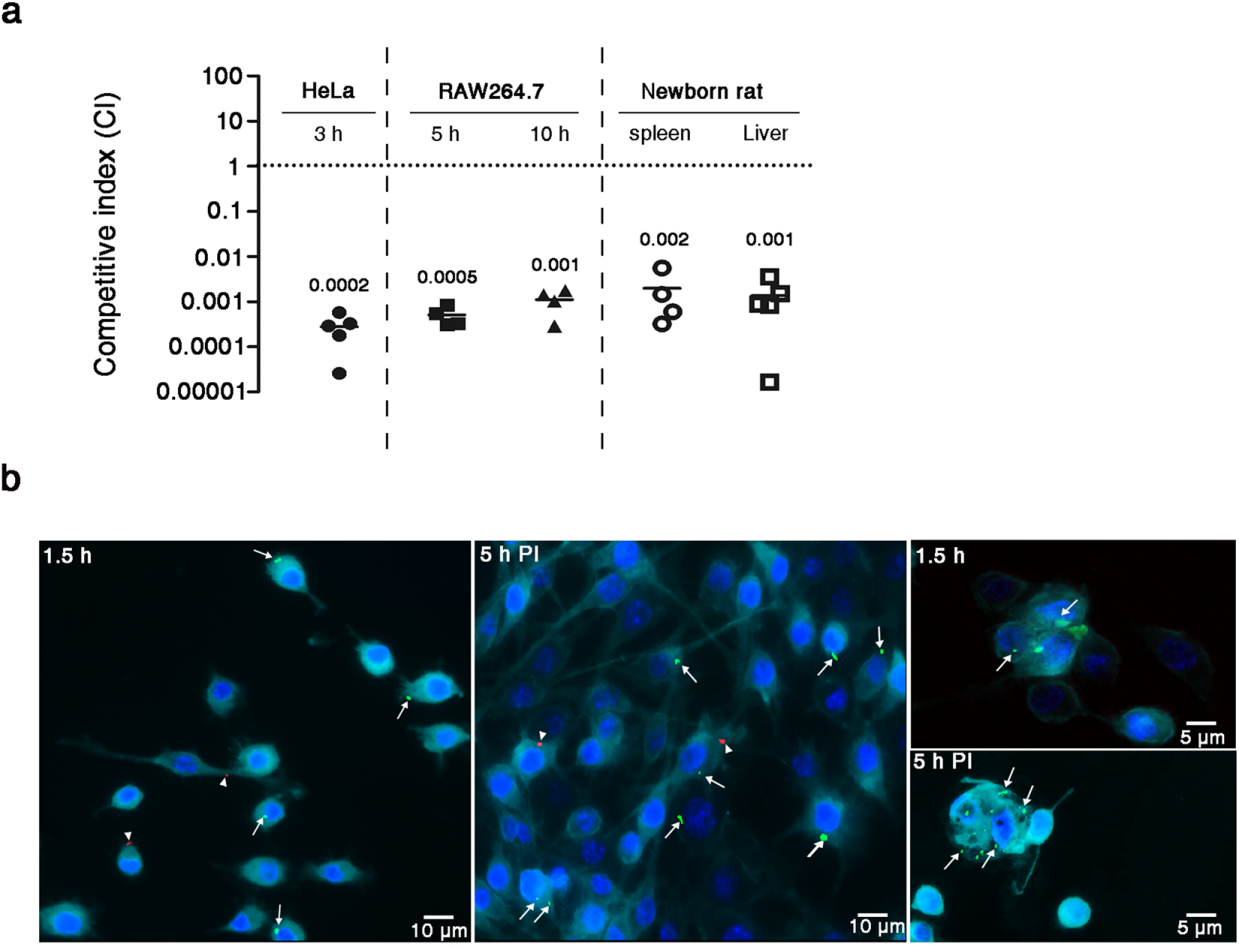
Absence of CSK29544_02616 reduces fitness in competition with WT during host infection. (**a**) Impaired fitness caused by the lack of CSK29544_02616. HeLa and RAW264.7 cells and rat pups (2–3 days old) were administered with a mixture of bacterial suspension containing equivalent CFUs of WT and ΔCSK29544_02616::*kan*^*R*^ strains. Bacterial fitness was assessed using CI. Mean CIs from indicated mammalian cells and five animals are presented above average lines. (**b**) Fluorescence microscopic analysis of RAW264.7 cells. Mammalian cells were infected with a bacterial mixture containing equivalent CFUs of WT expressing GFP (arrows) and ΔCSK29544_02616 expressing mCherry (arrow heads). At 1.5 h (phagocytosis) and 5 h post-infection, infected macrophage cells were stained with Hoechst 33342 (blue) and CellMask Deep Red (cyan) to detect the nucleus and plasma membrane, respectively. Scale bar: 5 or 10 μm.

The observation that ΔCSK29544_02616 was attenuated in both invasion and survival abilities indicates that CSK29544_02616 has various roles in the interaction of *C. sakazakii* with host cells. To better understand the behavior of ΔCSK29544_02616 cells during host infection, host cells were infected with a mixed culture of WT and ΔCSK29544_02616 strains, as the impaired virulence of some mutants may be complemented in *trans* by the presence of the WT strain in the inoculums. In both cases of epithelial cells and macrophages, the ΔCSK29544_02616 strain was significantly outcompeted by the WT strain (Fig. 2a). Microscopic analysis of macrophages co-infected with both strains revealed that the average numbers of intracellular bacteria per macrophage cell were 4- to 5-fold lower in the ΔCSK29544_02616 strain than in the WT strain (Fig. 2b and Supplementary Fig. S2). Under laboratory conditions used in this study, bacterial growth was comparable between the two strains (Supplementary Fig. S3). Taken together, these results demonstrate that the ΔCSK29544_02616 strain with attenuated invasion and survival abilities could not overcome its defects by co-infection with virulent bacteria.

The fitness of the ΔCSK29544_02616 strain in newborn rats was further assessed by co-infection with the WT strain^32,33^. The ΔCSK29544_02616 strain was outcompeted by the WT strain, showing competitive indices (CIs) of 0.002 and 0.001 in the spleen and liver, respectively, at 20 h post-infection (Fig. 2a). This result demonstrates that CSK29544_02616 is required for the survival and dissemination of *C. sakazakii* inside host animals.

### Loss of CSK29544_02616 gene results in alterations in outer membrane appendages

The CSK29544_02616 gene is conserved among *Cronobacter* species (Supplementary Fig. S4) and predicted to encode a hypothetical protein localized in the inner membrane or cytoplasm. Because the invasion of *C*. *sakazakii* into host cells is mediated by the interaction between host receptors and bacterial surface constituents^34^, we investigated whether deletion of CSK29544_02616 caused structural alterations in bacterial surface appendages including outer membrane proteins (OMPs), LPS, and flagella, which are known as key virulence determinants in *C. sakazakii* infection^6–8,35^.

**Figure 3:**
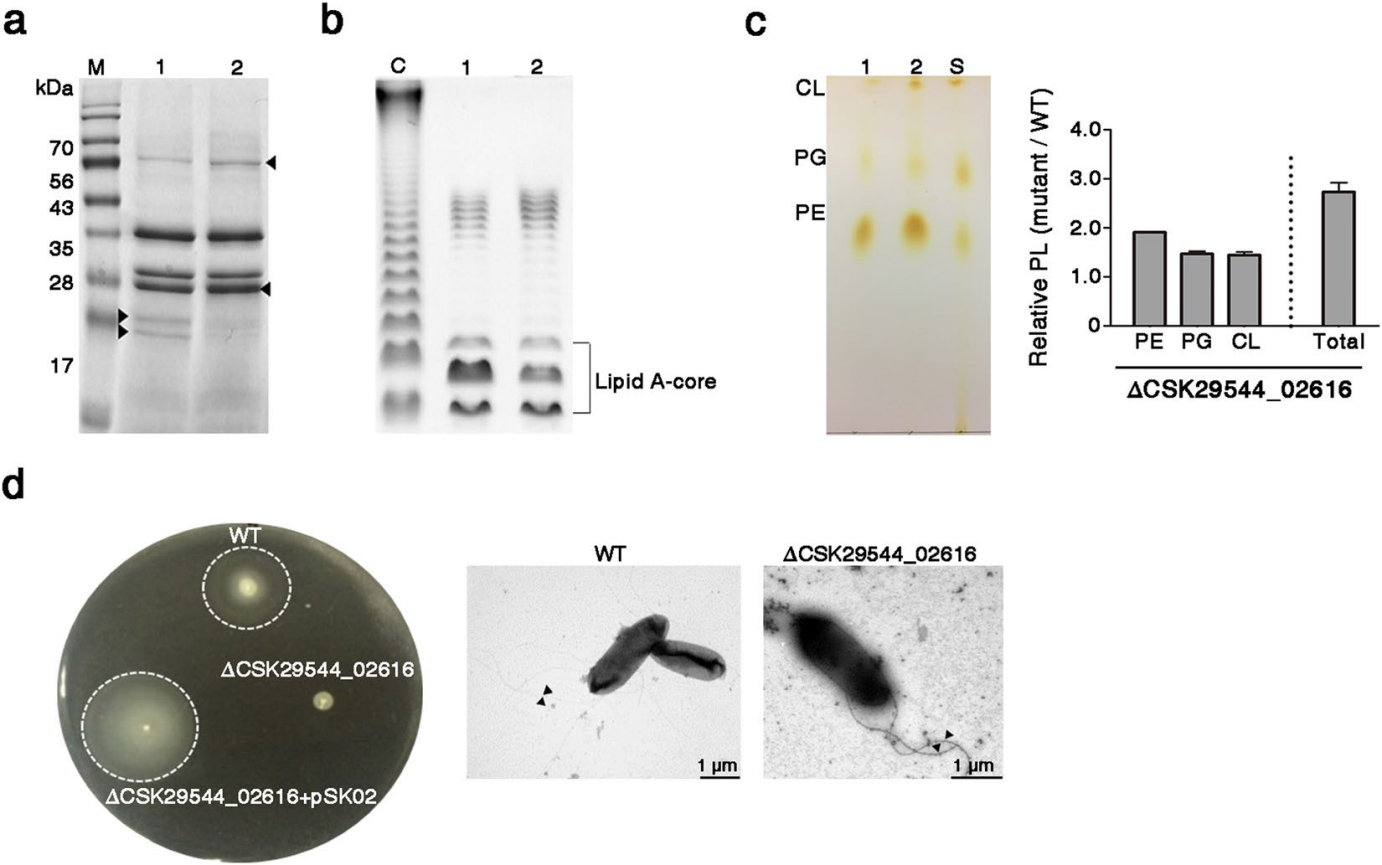
CSK29544_02616 influences the structural composition of the outer membrane. (**a**) Comparison of outer membrane protein profiles between WT (lane 1) and ΔCSK29544_02616 (lane 2) strains. Outer membrane fractions equivalent to 10 μg of proteins were analyzed by SDS-PAGE in parallel with molecular-weight (MW) size markers (M). Arrowheads indicate proteins with different abundance between the two strains. (**b**) LPS profiles in DOC-PAGE. LPS fractions were extracted by hot phenol-water method, separated on DOC-PAGE, and compared between WT (lane 1) and ΔCSK29544_02616 mutant (lane 2) strains. Lane C shows LPS from *Salmonella* Typhimurium LT2 as a positive control. (**c**) Increases in PL production in the absence of CSK29544_02616. Membrane PLs were extracted from equivalent bacterial cells of WT (lane 1) and CSK29544_02616 (lane 2) strains and separated by TLC analysis (left). Each constituent of membrane PL, separated by TLC analysis, and total membrane PLs before TLC analysis were quantified in duplicate using malachite green (right). Lane S indicates purified standard PLs, which were used to identify spots of each PL species. (**d**) Abolished motility caused by the loss of CSK29544_02616. *C. sakazakii* strains including WT, ΔCSK29544_02616, and the mutant complemented with pSK02 were injected into semi-solid agar plates (left panel). Plasmid pSK02 was designed to produce His-CSK29544_02616 protein using an arabinose-inducible promoter. Bacterial morphologies of WT and ΔCSK29544_02616 strains were analyzed by transmission electron microscopy. Arrowheads indicate flagella (right panel). Full-length images were represented in Supplementary Figure S10.

The protein profiles of the outer membrane fractions differed between WT and ΔCSK29544_02616 strains (Fig. 3a). *C*. *sakazakii* exploits OMPs such as OmpA, OmpX, and Inv for invading host cells^6,8,36^. However, the mRNA levels of *ompA*, *ompX*, or *inv* were not changed by the absence of CSK29544_02616 (Supplementary Fig. S5).

*C. sakazakii* LPS are known to facilitate bacterial invasion by disrupting the tight junction of epithelial cells^6^. Deoxycholate (DOC)-PAGE analysis showed that a lack of CSK29544_02616 decreased the abundance of lipid A and core oligosaccharide moiety without structural modification in LPS (Fig. 3b). Quantification of bacterial LPS by the purpald assay^37^ revealed an approximate 0.4 mM reduction in LPS levels by deletion of CSK29544_02616 (Supplementary Fig. S6). The outer membrane of gram-negative bacteria is an asymmetrical bilayer that is balanced with PL in the inner leaflet and LPS in the outer leaflet^38^. In the context of outer membrane biogenesis, bacteria maintain a proper balance in membrane constituents via regulatory crosstalk between the LPS- and PL- biosynthesis pathways^39^. Therefore, bacteria with defects in the LPS biogenesis pathway typically exhibit a compensatory increase in PL^40^. Accordingly, a decrease in LPS production resulted in a reciprocal increase of PL by 2.5-fold in the ΔCSK29544_02616 strain (Fig. 3c). The three main PLs including PE, PG, and CL were increased in the absence of CSK29544_02616.

Many bacterial pathogens utilize flagella not only as motile machinery but also as virulence determinants^41–43^. In this context, *C. sakazakii* applies flagella in adhesion onto intestinal epithelial cells and stimulation of pro-inflammatory cytokine production in macrophages^7,35^. The ΔCSK29544_02616 strain exhibited defective motility on 0.3% soft agar plates, but remained flagellated (Fig. 3d). The defective motility due to the loss of CSK29544_02616 was complemented by the introduction of pSK02, a plasmid expressing CSK29544_02616 under the control of an arabinose-inducible promoter (Fig. 3d).

### Alterations in outer membrane constituents by lack of CSK29544_02616 influence physiological properties of bacterial surface

**Figure 4:**
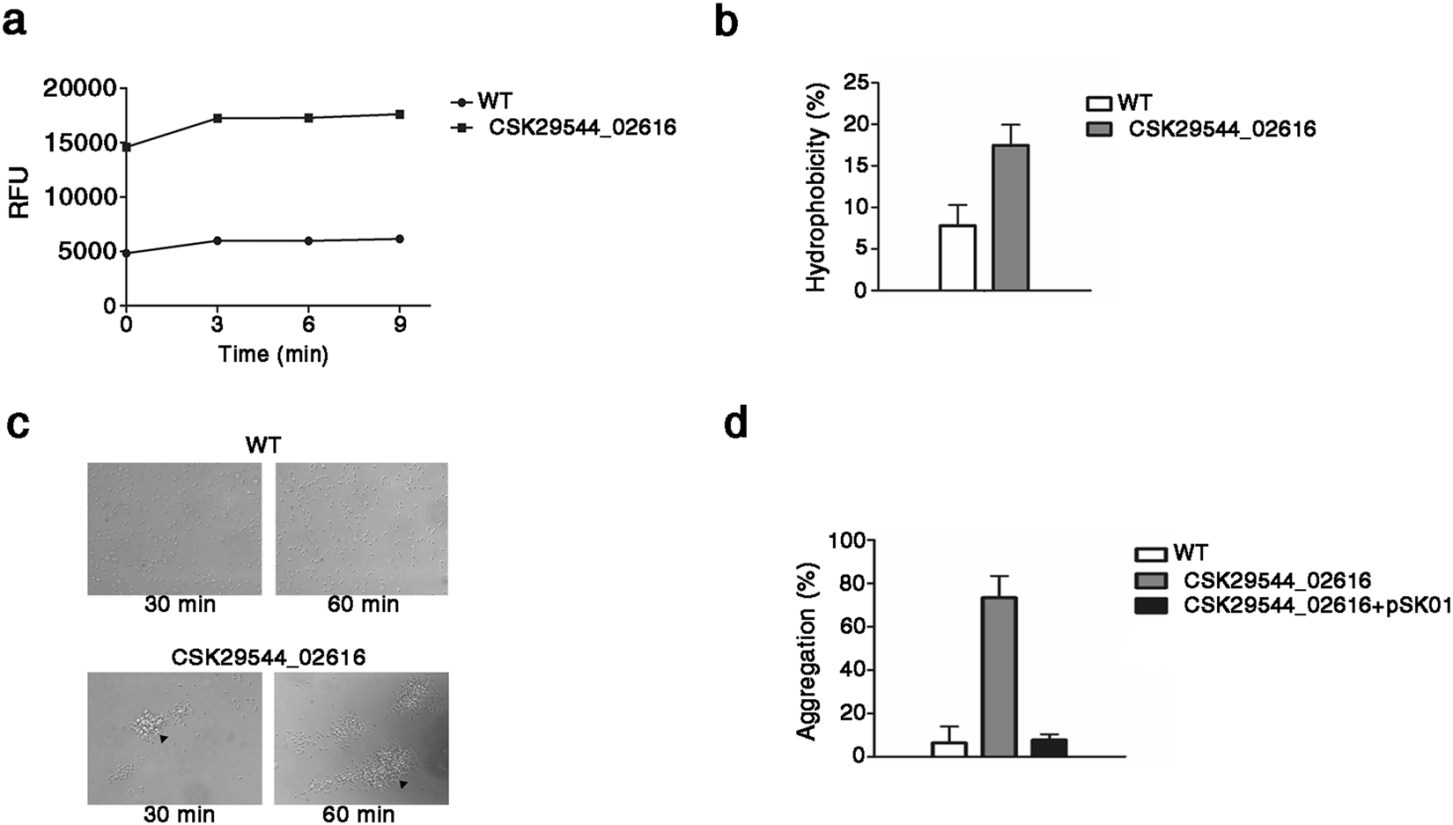
Loss of CSK29544_02616 increases hydrophobicity and permeability of cell surface, resulting in cell autoaggregation. (**a**) Assessment of impaired outer membrane integrity in the absence of CSK29544_02616. NPN uptake assay was performed in three independent biological replicates and depicted as RFU values. The graph was a representative of three replicates. (**b**) Elevated surface hydrophobicity in ΔCSK29544_02616. Hydrophobicity assay using xylene was conducted in triplicate in three independent tests. The following formula was used to calculate hydrophobicity: [(H_0_-H)/H_0_] × 100 (H_0_ and H, OD_600_ of bacterial suspension before and after xylene addition, see the details in Supplementary information). (**c**) Microscopic analysis of WT (upper panel) and ΔCSK29544_02616 (lower panel) strains in suspension. Bacterial suspensions containing comparable cell numbers of each strain were statically incubated for 30 min and 1 h and observed under a phase-contrast microscope. Arrowheads indicate cellular aggregation. Images are representatives of three different cover slips of the two strains. (**d**) Autoaggregation in the absence of CSK29544_02616. Bacterial cultures of WT, ΔCSK29544_02616, and ΔCSK29544_02616 harboring pSK01 strains were diluted at an OD_600_ of 1.0 using fresh LB broth and incubated statically at room temperature. Aliquots taken from the top of each bacterial suspension were subjected to OD_600_ measurement at the indicated time points to estimate cell density. The graphs represent results from three independent experiments conducted in triplicate. Aggregation (%) was estimated by the following formula: [(A_0_-A)/A_0_] × 100 (A_0_ and A, OD_600_ of bacterial suspension before and after static incubation, see the details in Supplementary information).

The lack of CSK29544_02616 led to tremendous structural changes in bacterial outer membrane components, including OMP, LPS, PL, and flagella. These changes may compromise the membrane structure integrity and impair bacterial virulence in contact with host cells. Particularly, migration of PL from the inner leaflet to the outer leaflet of the outer membrane may generate symmetric loci bilayered with PL, rendering the membrane permeable to hydrophobic antimicrobial compounds^44^. To measure the membrane integrity of the ΔCSK29544_02616 strain, bacterial cells were incubated with the hydrophobic fluorescent probe 1-phenylnaphthylamine (NPN), which only minimally intrudes the normal LPS-enriched outer membrane^45^. As expected, NPN uptake was increased by at least three-fold in the ΔCSK29544_02616 strain (Fig. 4a). Bacterial surface hydrophobicity was also increased by at least two-fold in the ΔCSK29544_02616 strain (Fig. 4b). Interestingly, microscopic observation revealed that *C. sakazakii* lacking CSK29544_02616 tended to show intercellular aggregation (Fig. 4c). An autoaggregation assay further confirmed the occurrence of rapid cell aggregation in the culture of the ΔCSK29544_02616 strain (Fig. 4d). Taken together, the lack of CSK29544_02616 caused extensive alterations in the bacterial surface structure, compromising the integrity and physiological properties.

### CSK29544_02616-encoded Labp specifically interacts with LpxA

**Figure 5:**
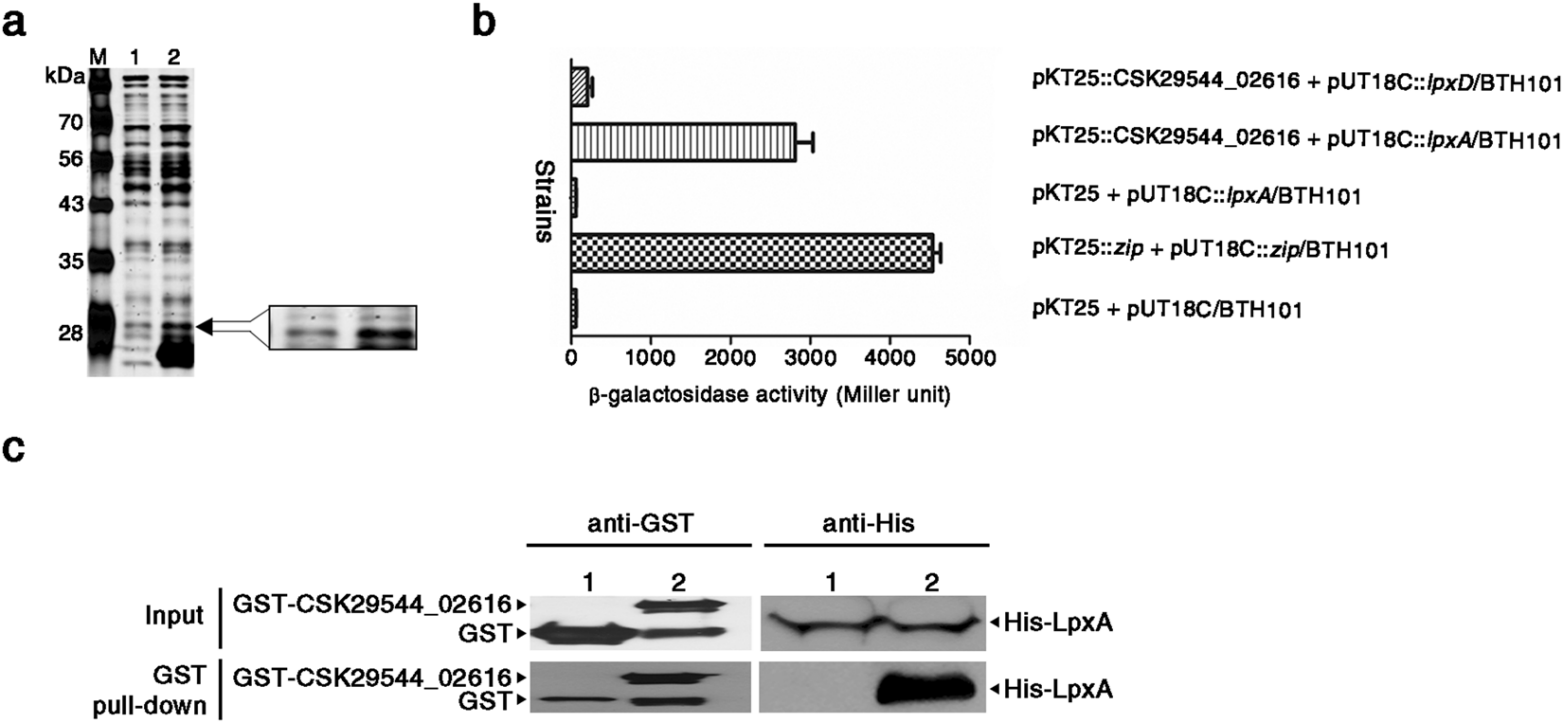
CSK29544_02616 specifically binds to LpxA, UDP-GlcNAc acyltransferase. (**a**) Ligand-fishing analysis with His-CSK29544_02616 as bait. Soluble cell extracts from ΔCSK29544_02616 were incubated in the absence (lane 1) or presence of His-CSK29544_02616 (lane 2) and passed through Ni-NTA resin. Eluted proteins along with MW size markers (M) were subjected to 12% SDS-PAGE. (**b**) Protein-protein interaction *in vivo* between CSK29544_02616 product and LpxA using a bacterial two-hybrid system. Plasmid pKT25 containing CSK29544_02616 and plasmid pUT18C harboring *lpxD* or *lpxA* were introduced into *E. coli* BTH101 reporter strain individually or in combination. The reporter strains were cultivated in LB broth supplemented with isopropyl-β-D-1-thiogalactopyranoside (IPTG). β-galactosidase activity was evaluated to determine protein-protein interactions between CSK29544_02616 product and LpxA. The experiment was performed in triplicate. (**c**) Protein-protein interaction *in vitro* between CSK29544_02616 and LpxA using GST pull-down analysis. *E. coli* ER2566 producing His-LpxA was mixed with *E. coli* BL21 (DE3) producing GST-CSK29544_02616 (lane 2) or GST alone (lane 1). Total cell lysates were incubated with glutathione (GSH)-agarose beads. Total lysates and GSH pulled-down fractions were subjected to immunoblotting using anti-GST and anti-His antibodies, respectively. Full-length gel and blot images were represented in Supplementary Figure S11.

The numerous changes in the bacterial surface structure by the lack of CSK29544_02616 indicates that CSK29544_02616 is involved in regulating fundamental constituents required for bacterial outer membrane biogenesis. To define the action mechanism of CSK29544_02616, ligand fishing was performed by using CSK29544_02616 as bait. To purify the CSK29544_02616 product, the CSK29544_02616 gene was modified to express a His_**×**6_-tag at its N-terminus (Supplementary Fig. S7b). Tagging CSK29544_02616 with His_**×**6_ did not hamper its intrinsic properties as demonstrated in the motility assay (Fig. 3d). A crude cell-free extract from the ΔCSK29544_02616 strain was incubated with the His_**×**6_-tagged CSK29544_02616 product (referred to as His-CSK29544_02616) and proteins bound to the bait protein were isolated using a metal-affinity chromatography column pre-equilibrated with Ni-NTA resin. One of these bound proteins at approximately 28 kDa (arrowhead in Fig. 5a) was matched with multiple proteins including LpxA by liquid chromatography (LC)-mass spectrometry (MS)/MS analysis (Supplementary Table S2). LpxA is a UDP-GlcNAc acyltransferase required for the first step of lipid A biosynthesis. We validated the specific interaction between CSK29544_02616 protein and LpxA *in vivo* using the bacterial two-hybrid system based on adenylate cyclase reconstitution (Fig. 5b) and confirmed the results *in vitro* using a GST pull-down assay (Fig. 5c). LpxD is similar to LpxA because they both are acetyltransferases that can incorporate the *R*-3-hydroxymyristate moiety into the lipid A precursor^46^. However, LpxD did not interact with CSK29544_02616 protein (Fig. 5b), indicating LpxA-specific recognition by CSK29544_02616. Therefore, the CSK29544_02616 gene was designated as an LpxA-binding protein (*labp*) gene.

### Labp improves enzymatic activity of LpxA via direct interaction

**Figure 6:**
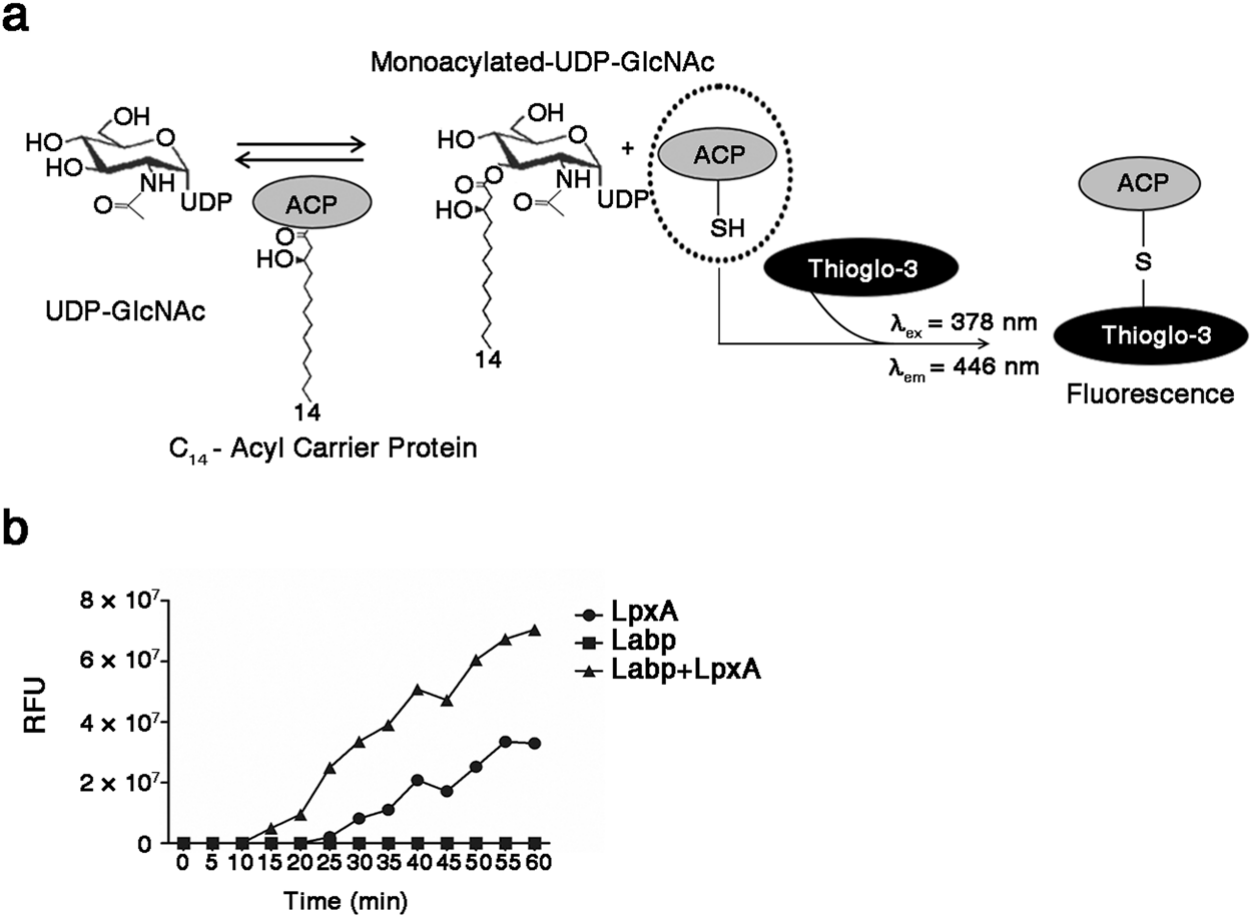
Labp encoded by CSK29544_02616 improves LpxA enzymatic activity via direct interaction. (**a**) A schematic representation of LpxA enzymatic assay. The acyl-group transfer to UDP-GlcNAc by LpxA generates holo-ACP with an exposed single free-thiol, which is in turn immediately conjugated with ThioGlo-3, a thiol-specific labeling reagent, leading to fluorescence at λ_ex_ = 378 nm and λ_em_ = 446 nm. (**b**) Increased LpxA catalytic activity in the presence of Labp. LpxA and Labp were added to a mixture of UDP-GlcNAc and *R*-3-hydroxymyristoyl-ACP individually or in combination and the fluorescence of ThioGlo-ACP conjugates was depicted as the RFU value. Data are representative of five independent assays.

The interaction of Labp with LpxA prompted us to examine whether Labp influences the catalytic activity of LpxA during lipid A biosynthesis. It is known that LpxA transfers the *R*-3-hydroxyacyl chain from ACP to the 3-hydroxyl group of UDP-GlcNAc and that its catalytic activity can be measured using a fluorescence reagent, ThioGlo-3, which senses the presence of holo-ACP deprived of an acyl group (Fig. 6a)^47^. The addition of LpxA into a mixture of substrates of UDP-GlcNAc and *R*-3-hydroxymyristoyl-ACP produced fluorescent ThioGlo-ACP conjugates (Fig. 6b and Supplementary Fig. S8a). Interestingly, co-application of Labp and LpxA in the substrates approximately doubled the fluorescence of ThioGlo-ACP conjugates. However, the addition of Labp alone did not result in any fluorescence (Fig. 6b), suggesting that Labp is an activator in the LpxA-mediated reaction. The combination of LpxA with bovine serum albumin did not improve the enzymatic activity of LpxA. Instead, it dampened fluorescence production, corroborating the favorable interaction between LpxA and Labp (Supplementary Fig. S8b).

**Figure 7:**
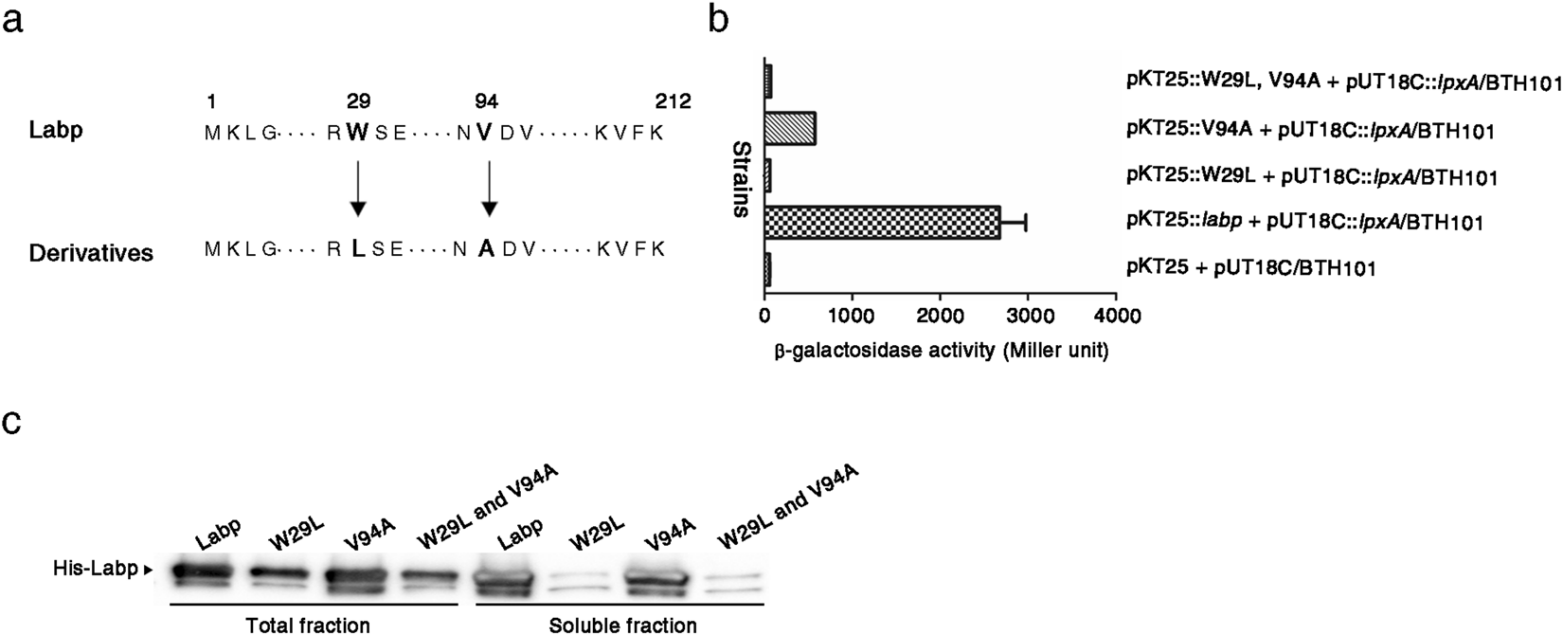
Valine 94 of Labp is a key residue in its specific interaction with LpxA. (**a**) Substitution strategy of amino acids in modified Labp proteins. Tryptophan residue 29 and valine residue 94 were substituted with lysine and alanine (bold letters), respectively, using site-directed mutagenesis. (**b**) Compromised interaction between Labp V94A and LpxA. *E. coli* BHT101 harboring pUT18C::*lpxA* was transformed with pKT25 plasmids to produce Labp or its derivatives (W29L and V94A). The interaction between LpxA and Labp derivatives was evaluated as described for the bacterial two-hybrid assay. The experiment was performed in triplicate and average values were plotted. (**c**) Comparison of protein-production levels between Labp and its derivatives (W29L and V94A). Total cell lysates and soluble lysates prepared from bacteria producing Labp or its derivatives were resolved by SDS-PAGE and subjected to immunoblotting using antibodies specific for the His epitope tag. Full-length blot image was represented in Supplementary Figure S12.

It has been reported that LpxA forms a homotrimer that possesses three active sites in the interfaces of adjacent subunits^48^. Labp binding to LpxA may induce advantageous structural conformations for LpxA to exert catalytic activity to produce UDP-3-(*R*-3-hydroxyacyl)-GlcNAc. To understand the catalytic mechanism of the LpxA/Labp complex, mutagenesis studies were conducted on Labp. *labp* was amplified using an error-prone PCR polymerase and PCR products were expressed in a bacterial two-hybrid system in combination with intact *lpxA*. *E. coli* clones showing decreased interactions between LpxA and Labp derivatives revealed mutations at both residues of tryptophan (W) at 29 (W29) and valine (V) at 94 (V94) in amino acid sequences of Labp. To define critical amino acids responsible for the interaction of Labp with LpxA, W29 and V94 in Labp were substituted with leucine (L) and alanine (A), respectively (Fig. 7a). The binding affinity of such Labp derivatives toward LpxA was then evaluated in the bacterial two-hybrid assay (Fig. 7b). Labp V94A caused a reduction in ß-galactosidase activity (by approximately four-fold) compared to intact Labp, indicating the importance of the V94 residue in the interaction of Labp with LpxA. The replacement of tryptophan at 29 with leucine markedly decreased the protein levels of Labp in whole-cell lysates and soluble fractions (Fig. 7c) and Labp W29L showed minimal ß-galactosidase activity in the bacterial two-hybrid assay (Fig. 7b). Labp W29L may have an unfavorable conformation that is vulnerable to proteolytic processes or may be prone to aggregate, making it insoluble (Fig. 7c). Alternatively, according to their predicted outwardly exposed locations, mutations at W29 and V94 may prevent Labp from interacting with LpxA (Supplementary Fig. S9).

## Discussion

In this study, CSK29544_02616 (*labp*) was defined as a new virulence gene in *C. sakazakii*. Mutant strains lacking *labp* were defective in invasion into epithelial cells, persistence inside macrophages, and dissemination into the spleen and liver of host animals. Despite the presence of the flagellar structure on the bacterial surface, motility of the Δ*labp* mutant was deteriorated (Fig. 3d). Pathogenic bacteria with functional flagellar machinery can gain access to layers of epithelial cells with a higher probability than motility-defective cells. In this context, the compromised motility of the Δ*labp* mutant may decrease the invasion ability into epithelial cells (Fig. 1). Additionally, the Δ*labp* mutant was prone to aggregate and form clumps, likely because of its increased surface hydrophobicity (Fig. 4b). This morphological change may disturb macrophage phagocytosis of the Δ*labp* mutant strain (Fig. 1). Macrophages phagocytose bacterial invaders with dimensions of 2–3 μm, the general size of a single bacterium, but recognizes very few larger particles^49^. Extensive phenotypic alterations by the *labp* deletion suggested that the attenuated virulence of the Δ*labp* mutant strain occurred because of a malfunction in lipid A biosynthesis considering the profound impact of LPS on bacterial infection to host animals. Labp directly bound to LpxA and stimulated its catalytic activity (Figs 5 and 6) and its absence caused a clear reduction in lipid A production (Fig. 3b and Supplementary Fig. S6). *C*. *sakazakii* intrudes the intestinal epithelium, the protective barrier against luminal bacterial pathogens, and its LPS play a key role in disrupting tight junctions in the epithelial cell monolayers^8,34^. Therefore, a mutant with compromised production of lipid A or LPS may fail to invade the layers of epithelial cells. Furthermore, *C. sakazakii* with a low LPS abundance may show lower phagocytosis by macrophages and disturb inflammatory responses in macrophages^50^.

The lipid A biosynthesis pathway in *E. coli* involves nine enzyme-catalyzed steps. It requires UDP-GlcNAc and *R-*3-hydroxymyristoyl-ACP as starting materials. However, according to the crosstalk models of lipid A, phospholipid, and peptidoglycan biogenesis, two substrates of UDP-GlcNAc and *R-*3-hydroxymyristoyl-ACP in lipid A biosynthesis are also associated with the other two metabolic pathways: UDP-GlcNAc is utilized as a substrate in peptidoglycan synthesis^24^, while *R-*3-hydroxymyristoyl-ACP is involved in the phopholipid biosynthesis pathway^15,51,52^. Therefore, a malfunction in LpxA, the first enzyme that consumes both substrates for the production of UDP-3-(*R*-3-hydroxymyristoyl)-GlcNAc in the lipid A biosynthesis pathway, may cause constitutional imbalance in the envelope structure, leading to disadvantageous physiological changes. We observed that the Δ*labp* mutant exhibited multiple phenotypic characteristics attributable to LpxA malfunction, including increased PL production, increased surface hydrophobicity, and cellular aggregation.

LpxA acylates UDP-GlcNAc with *R-*3-hydroxymyristoyl-ACP. Its equilibrium constant (*K*_eq_) is approximately 0.01 *in vitro*, indicating an unfavorable reaction that is readily revertible back into the substrate direction^25^. However, a thermodynamically unfavorable reaction of LpxA is unusual in that the thioester moiety of *R-*3-hydroxyacyl-ACP is energy-rich and acyl transfer from ACP to the 3-OH group of UDP-GlcNAc is favorable^25^. LpxA forms a homotrimeric structure and locates the donor substrate *R-*3-hydroxyacyl-ACP at the interfaces of adjacent subunits near the three active sites^48^. Binding of Labp to LpxA may aid in the long hydrophobic *R-*3-hydroxyacyl chain of the donor substrate to become positioned near LpxA’s active sites so that it can be committed to the recipient substrate UDP-GlcNAc. Alternatively, the interaction between LpxA and Labp may induce conformational changes in LpxA bound to the product UDP-3-(*R-*3-hydroxyacyl)-GlcNAc, enabling the reaction product to proceed through the lipid A biosynthesis pathway rather than reverting into substrates. Further investigations are required to determine how Labp exerts its positive role in the lipid A biosynthesis of LpxA.

Considering that *labp* is conserved among *Cronobacter* species, it may be useful as a target for controlling *Cronobacter* infection. Determining the mechanism of action of Labp involved in stimulation of LpxA activity will improve our understanding of the physiological and pathogenetic characteristics of *Cronobacter* spp. and aid in the design of new approaches for controlling *C. sakazakii* infection.

## Methods

### Bacterial strains and plasmids

All bacterial strains and plasmids used in this study are listed in Supplementary Table S1. Details of strain and plasmid construction are provided in the supplementary information.

### Invasion assay

To examine the bacterial invasion ability into mammalian Caco-2 cells, a gentamicin protection assay was performed as described previously^53^. Briefly, mammalian Caco-2 cells were seeded into 24-well cell culture plates at a density of 2 × 10^5^ cells per well, supplemented with Eagle’s minimum essential medium (EMEM) (ATCC) containing 20% fetal bovine serum (FBS) (Gibco, USA), and incubated for one day before bacterial infection. Bacteria were grown overnight, transferred to fresh LB medium at 1%, and incubated at 37 °C for 2.5 h with constant shaking. Next, *C. sakazakii* cells were collected by centrifugation at 20,000 × *g* for 1 min, washed with phosphate-buffered saline (PBS) (pH 7.4), and resuspended in 1 mL of pre-warmed fresh EMEM. Caco-2 cell monolayers were infected with bacteria at a multiplicity of infection (MOI) of 100 and incubated for 1.5 h in the presence of 5% CO_2_. After washing with PBS, cells were further incubated in fresh medium containing gentamicin (100 μg/mL) for 1.5 h followed by three washes with PBS. Cells were then lysed with 1% Triton X-100 (500 μL/well). Cell lysates were serially diluted and plated onto LB agar to count the number of intracellular bacteria.

### Survival assay

A long-term survival assay was performed as described previously^53^. Briefly, RAW264.7 murine macrophage-like cells were seeded into 24-well cell culture plates at a density of 5 × 10^5^ cells per well and incubated in Dulbecco Modified Eagle Medium (DMEM) supplemented with 10% FBS for 1 day at 37 °C with 5% CO_2_. *C. sakazakii* strains were prepared as described for the invasion assay and added to monolayers of RAW264.7 murine macrophage-like cells at an MOI of 100. After incubation at 37 °C for 45 min, the medium was replaced with fresh DMEM containing gentamicin (100 μg/mL) and incubated for an additional 45 min. Intracellular bacteria were enumerated in a similar manner to estimate the level of bacterial internalization by macrophages. To evaluate long-term persistence inside macrophages, the cells were replenished daily with fresh medium containing gentamicin (10 μg/mL) and subjected to lysis at 24, 48, and 72 h after incubation. Counting of live bacterial cells inside macrophages was performed.

### LPS extraction

LPS were extracted from bacteria grown overnight using a hot phenol-water micro-extraction method^54,55^. Bacterial cells from 1 mL of culture (approximately 2 × 10^9^ CFU/mL) were harvested, washed once with 1 mL of DPBS (Dulbecco’s PBS containing 0.15 mM CaCl_2_ and 0.5 mM MgCl_2_), and re-suspended in 100 µL of DPBS. After bacterial cell disruption by sonication, the cell lysate was treated with proteinase K at a concentration of 100 µg/mL and incubated at 37 °C for 1 h followed by the addition of ddH_2_O. An equivalent volume of a preheated (68 °C) phenol solution was added to the cell lysate and the mixture was incubated at 68 °C for 15 min with vigorous vortexing every 5 min. The sample was chilled on ice for 5 min and the aqueous phase was separated by centrifugation at 10,000 × *g* for 5 min at 4 °C. LPS were extracted again from the remaining phenol phase with an additional ddH_2_O addition. The pooled aqueous phase was subsequently mixed with 10 volumes of 95% ethanol and sodium acetate (at final concentration of 0.5 M) and incubated overnight at −20 °C. Crude LPS were pelleted by centrifugation at 16,000 × *g* for 5 min at 4 °C, re-suspended in 100 µL of ddH_2_O, and precipitated again with 95% ethanol. The precipitated LPS were then re-dissolved in 50 µL ddH_2_O and stored at −20 °C. The extracted LPS fraction was subjected to DOC-PAGE using 15% acrylamide gels^56^. These gels were fluorescently stained using the Pro-Q Emerald 300 Lipopolysaccharide Gel Stain Kit (Molecular Probes, USA) according to the manufacturer’s instructions. LPS samples were visualized under UV at a wavelength of 300 nm using a Gel DocTM EZ imager (Bio-Rad, USA).

### LPS quantification

Quantification of bacterial LPS was performed as described previously^37^. Briefly, an aliquot of the extracted LPS (25 µL) was mixed with 50 µL of 32 mM NaIO_4_ (Sigma, USA) in a 96-well plate. After incubation for 25 min, each well in the plate was treated with 50 µL of 136 mM purpald reagent in 2 N NaOH (Sigma, USA) and 50 µL of 64 mM NaIO_4_. After incubation for another 20 min, the absorbance of each well was measured with a Synergy HTX multi-mode reader (BioTek, USA) at 550 nm. To construct a standard curve to calculate the molarity of LPS, Kdo (2-keto-3-deoxyoctonate) (Sigma, USA) was used at different concentrations.

### Lipid extraction and TLC analysis

Lipid extraction was conducted as previously described^57^. Briefly, bacterial cells grown exponentially were washed twice with PBS and resuspended in 1.9 mL of PBS. Bacterial cell suspensions were mixed with 2.4 mL of chloroform and 4.8 mL of methanol to generate a single-phase Bligh and Dyer mixture, incubated at room temperature for 30 min, and subjected to centrifugation at 1500 × *g* for 20 min. The supernatant was converted into a two-phase solution by adding 2.4 mL of PBS and 2.4 mL of chloroform and centrifuged at 1500 × *g* for 20 min. The lower phase was removed and dried under a stream of nitrogen gas. For thin-layer chromatography (TLC) to analyze phospholipid, the dried lipid extracts were dissolved in 100 μL of a mixture of chloroform-methanol (2:1). Next, 5 μL of each sample was spotted onto a TLC silica gel 60 plate and the plate was developed in a tank equilibrated with chloroform-methanol-acetic acid (70:20:10 [v/v/v]). After drying the plate, lipids were visualized using iodine vapor. For spot identification, phosphatidylethanolamine (PE) (Sigma, USA), phosphatidylglycerol (PG) (Sigma, USA), and diphosphatidylglycerol (DPG) (Sigma, USA) were spotted onto TLC plate in parallel. After identifying the spots, the spots were scraped and subjected to digestion with perchloric acid. Each PL was quantified by malachite green staining as previously described^58^.

### Outer membrane fraction

Bacteria cells were grown in LB broth overnight, transferred to fresh LB medium at 1%, and incubated until they reached the mid-log phase of growth. These cells were collected by centrifugation at 10,000 × *g* for 5 min and resuspended in 10 mL of HEPES buffer (10 mM, pH 7.4). The resuspension was ultra-sonicated, centrifuged, and filtered with a 0.22-μm filter membrane. The membrane fraction was collected by ultra-centrifugation at 100,000 × *g* for 1 h at 4 °C. The pellet was washed with 10 mL of 10 mM HEPES (pH 7.4) and ultra-centrifuged again as described above. The pellet was resuspended in 10 mL of 10 mM HEPES (pH 7.4) containing 2% *N-*lauroylsarcosine (wt/vol) (Sarkosyl) (Sigma, USA) and incubated at 37 °C for 30 min with shaking. Sarkosyl-treated membranes were centrifuged at 100,000 × *g* for 1 h at 4 °C and the pellet was resuspended in 50 mM Tris-Cl (pH 8.0) containing 1% (wt/vol) Zwittergent 3–14 (Calbiochem, USA) and 10 mM EDTA.

### Outer membrane permeability assay

An NPN (Sigma, USA) access assay was conducted as described previously^59^. Briefly, bacterial cells were grown in LB broth overnight, transferred to fresh LB medium at 1%, and incubated until they reached the mid-log phase of growth. Cells, adjusted to OD_600_ of 0.1, were harvested and suspended in 10 mM HEPES (pH 7.4) buffer. NPN was added to a final concentration of 1 µM. Fluorescence was measured continuously at λ_ex_ = 350 nm and λ_em_ = 420 nm for 15 min at 3-min intervals. Relative fluorescence unit (RFU) values were normalized to the numbers of viable cells used in the assay.

### Ligand fishing to search for proteins that interact with His-CSK29544_02616

*C. sakazakii* SK015 lacking CSK29544_02616 was used to isolate CSK29544_02616 protein tagged with six histidines at the N-terminus. The cell lysate of ΔCSK29544_02616 was mixed with 1 mg of His-CSK29544_02616 and incubated at 4 °C for 1 h with gentle end-over-end rotation in a Poly-Prep chromatography column (Bio-Rad, USA) pre-equilibrated with Ni-NTA resin (Qiagen, Germany). After 1 h of incubation, each column was washed five times with 1 mL of washing buffer (20 mM Tris-Cl containing 300 mM NaCl and 10 mM imidazole, adjusted to pH 8.0). Proteins bound to the column were eluted with elution buffer (20 mM Tris-Cl containing 300 mM NaCl and 250 mM imidazole, adjusted to pH 8.0). Aliquots of eluted protein samples were subjected to SDS-PAGE and Coomassie brilliant-blue R staining. Proteins that specifically bound to His-CSK29544_02616 were cut from the gel, treated with trypsin using an in-gel digestion method, and subjected to peptide-mapping analysis by the Yonsei Proteome Research Center (Korea) as described in the LC-MS/MS analysis^60^.

### Bacterial adenylate cyclase-based two-hybrid system (BACTH)

Protein-protein interactions were verified by reconstituting adenylate cyclase (CyaA) in *E. coli* through hetero-dimerization of fusion proteins^61^. The *E. coli* reporter strain BTH101 harboring bait and prey plasmids was used to evaluate the protein-protein interaction between CSK29544_02616 and LpxA. To quantify the binding affinity between the two proteins, a β-galactosidase assay was performed. The results are expressed in Miller units^62^. BTH101 strains harboring pKT25-zip/pUT18C-zip and pKT25/pUT18C were used as positive and negative controls, respectively.

### *In vitro* GST pull-down assay

His-LpxA and GST-CSK29544_02616 were produced in *E. coli* ER2566 and BL21 (DE3) cells, respectively, by adding 0.5 mM isopropyl β-D-1-thiogalactopyranoside (IPTG). Equivalent numbers of bacterial cells from these two strains were mixed at a 1:1 ratio in IP150 buffer (50 mM Tris-HCl at pH 7.4, 150 mM NaCl, 2 mM MgCl_2_, and 0.1% NP-40) containing a protease-inhibitor cocktail (Sigma, USA). These cells were disrupted by ultra-sonication. Cellular debris was removed by centrifugation at 21,130 × *g* for 1 h at 4 °C. Protein concentration in the supernatant was measured using the Bradford assay. Protein sample (1 mg) was incubated with 20 μL of GSH-agarose beads at 4 °C with gentle end-over-end rotation for 3 h. After washing three times with IP150 buffer, proteins bound to the beads were eluted through boiling in Laemmli’s SDS sample buffer (GeneDEPOT, USA) for 10 min. Protein samples were then subjected to SDS-PAGE followed by immunoblotting using antibodies specific for GST and His epitope tags.

### Fluorescent enzyme assay for LpxA and CSK29544_02616

To determine LpxA activity, a fluorescent enzyme assay was conducted as described previously^47^ with a few modifications. Briefly, all substrates used in the assay were dissolved in 20 mM HEPES (pH 8.0) in a final volume of 100 μL. A SpectraMax i3 plate reader (Molecular Devices, USA) was used to monitor fluorescence. Photomultiplier tube sensitivity was set to a low value to prevent saturation. The number of readings was set to 40. Substrates of 3-hydroxymyristoyl-ACP and UDP-GlcNAc were added to each well of 96-well optical bottom plates (Thermo Fisher Scientific, USA) containing 10 μL of 100 μM ThioGlo3 (COVALENT Associates, Inc., USA) at 8 μM and 4 mM, respectively. After incubation in the dark at 30 °C for 40 min to allow unacylated ACP to react with ThioGlo solution, either 10 nM of LpxA solution or 300 nM of His-CSK29544_02616 was added directly to the wells and mixed gently before measurement. The plate was read continuously at λ_ex_ = 378 nm and λ_em_ = 446 nm for 2 h at 15-s intervals. Reactions that included all substrates and reagents without LpxA or His-CSK29544_02616 were used as negative controls, while reactions containing all substrates, reagents and LpxA but without His-CSK29544_02616 were used as positive controls.

### Fluorescence microscopic analysis

RAW264.7 murine macrophage-like cells were seeded onto 12-mm glass cover slips (Thermo Fisher Scientific, USA) in 24-well cell culture plates at a density of 5 × 10^5^ cells per well and incubated in DMEM supplemented with 10% FBS in the presence of 5% CO_2_ for one day. RAW264.7 cells were co-infected with a 1:1 mix of a WT strain producing GFP (SK016) and ΔCSK29544_02616 strain producing mCherry (SK017). After incubation at 37 °C for 45 min, the medium was replaced with fresh DMEM containing gentamicin (100 μg/mL) and incubated for an additional 45 min. The coverslips were washed with PBS three times and treated with 4% paraformaldehyde for 10 min. Fixed cells were washed again with PBS and treated with a mixture of Hoechst (Thermo Fisher Scientific, USA) and CellMask Deep Red (Thermo Fisher Scientific, USA) for 30 min. After washing the coverslips with PBS, they were analyzed by DMi8 Microscope (Leica, Germany).

### *In vivo* animal study

Two- or three-day-old Sprague-Dawley (SD) rat pups were used to assess the virulence of WT and CSK29544_02616::*kan* (SK010) strains. Bacterial cells at mid-log phase of growth were collected, washed, and resuspended in PBS. For the competitive assay, cells of the WT and SK010 strains were mixed at 1:1 ratio and administered orally to rat pups at 2 × 10^9^ CFU/rat. To analyze bacterial colonization in organs, all rat pups were sacrificed by CO_2_ at 20 h post-infection. The spleens and livers were aseptically removed, homogenized, serially diluted, and plated onto LB agar in the presence or absence of kanamycin. CI was estimated using the following formula: (No. of SK010_output_/No. of SK010_input_)/(No. of WT_output_/No. of WT_input_)^12^.

### Ethical approval

The animal work in this study was approved by the Seoul National University Institutional Biosafety Committee (SNUIBC) under the permit number SNUIBC-P140306-1. Animal experiments were conducted in strict accordance with the guide provided by the Seoul National University Institutional Animal Care and Use Committee (SNUIACUC).

### Data Availability

All data generated during the current study are available from the corresponding author upon reasonable request.

## Electronic supplementary material

Supplementary Information
