# Supplementary material for: New virulence factor CSK29544_02616 as LpxA binding partner in Cronobacter sakazakii
Source: Sci Rep. 2018 Jan 16;8:835. doi: 10.1038/s41598-018-19306-0 (PMC5770445; doi:10.1038/s41598-018-19306-0)
Supplement: Supplementary file 1 — Supplementary Information [file 41598_2018_19306_MOESM1_ESM.pdf]

Supplementary information

New virulence factor CSK29544_02616 as LpxA binding partner in *Cronobacter sakazakii*

Seongok Kim[1,2], Hyunjin Yoon[2*], and Sangryeol Ryu[1*]

[1]Department of Food and Animal Biotechnology, Department of Agricultural Biotechnology, Research Institute for Agriculture and Life Sciences, and Center for Food and Bioconvergence, Seoul National University, Seoul 08826, Korea.

[2]Department of Molecular Science and Technology, Department of Applied Chemistry and Biological Engineering, Ajou University, Suwon 16499, South Korea.

***** Correspondence and requests for materials should be addressed to Sangryeol Ryu (email: sangryue@snu.ac.kr) and Hyunjin Yoon (email: yoonh@ajou.ac.kr). These authors contributed equally.

Supplementary Information

Materials and Methods

Construction of bacterial strains

Strains used in this study are listed in Supplementary Table S1. The complete genome sequence of *C. sakazakii* ATCC 29544 (Genbank accession No. CP011047) was used to design all PCR primers used in this study. Site-specific mutagenesis of *C. sakazakii* ATCC 29544 was performed according to the method of Dasenko and Wanner.[1] Briefly, kanamycin-resistance cassette from plasmid pKD13 was amplified using the following primers: CSK29544_02616-lamb-F (5'-CGC GTG GTG GAT GCT ATC CGC GCG CAG GCC GCG CTC GGC CTT GCG GAG AAA GTG GCA TGA TGT AGG CTG GAG CTG CTT CG-3') and CSK29544_02616-lamb-R (5'-CGT CAT TGA CTA CTG CCG CTC CCA TGC CAA CGA TAG CGT CGT TAC CGA TGT GGA TTT GCT ATT CCG GGG ATC CGT CGA CC-3'). PCR products were introduced into *C. sakazakii* ATCC 29544 harboring pKD46 plasmid by electroporation. Recombinant cells were recovered on Km-containing agar plates. The kanamycin-resistance cassette was removed from the chromosome of recombinant clones using pCP20 plasmid as described by Datsenko and Wanner.[1] Primers used for strain construction are listed in Table S3.

Construction of random transposon-mutant libraries and screening

Random mutagenesis was performed using EZ-Tn5^{TM} pMOD-2<MCS> transposon system (Epicentre, USA) as described previously.[2] Briefly, DNA fragments of EZ-Tn5 transposon were isolated by PvuII restriction enzyme digestion of the pMOD-2<MCS> and introduced into *C. sakazakii* ATCC 29544 by electroporation at 1.8 kV (Bio-Rad, USA). Transformants were selected on tryptic soy agar plates containing kanamycin (50 μg/ml). Transposon mutants were individually cultured and stored at - 80°C in TSB containing 15% (vol/vol) glycerol.[2]

Determination of transposon-insertion sites

To locate transposon-insertion sites in recombinant clones with attenuated invasion to host cells, their genomic DNAs were isolated, fragmented with restriction enzyme (EcoRV), and subjected to self-ligation according to the manufacturer's protocol (Epicentre, USA). Circularized DNA mixture was PCR-amplified and sequenced with Tn5-specific primers provided by the manufacturer (pMOD<MCS> forward-sequencing primer and pMOD <MCS> reverse-sequencing primer).

Construction of recombinant plasmids

All plasmids used in this study are listed in Table S1. To generate pSK01 producing CSK29544_02616 under its putative intrinsic promoter, CSK29544_02616 gene containing its upstream DNA sequences was PCR-amplified using primers of

CSK29544_02616-F-SphI and CSK29544_02616-R-BamHI. PCR products were introduced into pACYC184 digested with SphI and BamHI. For the construction of pSK02 expressing His-CSK29544_02616 under arabinose-inducible promoter, CSK29544_02616 was PCR-amplified with His-CSK29544_02616-F and His-CSK29544_02616-R primers and PCR products were inserted into pBAD24 cut with EcoRI and SalI. CSK29544_02616, *lpxA*, and *lpxD* genes were amplified using primers of CSK29544_02616-F-BamHI and CSK29544_02616-R-EcoRI, lpxA-F-SalI and lpxA-R-BamHI, and lpxD-F-SalI and lpxD-R-BamHI, respectively. These PCR products were inserted between BamHI and EcoRI sites of pKT25 vector or SalI and BamHI sites of pUT18C vector. In the construction of pSK05 and pSK06 for GST pull-down assay, pETDuet-1 and pGST parallel 1 vectors were used to express His-LpxA and GST-CSK29544_02616, respectively. Plasmids pSK07, pSK08, and pSK09 were generated for protein purification and used for LpxA enzymatic assay. Plasmids expressing CSK29544_02616 derivatives with W29L and V94A substitutions were constructed using the following primers: CSK29544_02616-W29L-F and CSK29544_02616-W29L-R for CSK29544_02616 W29L, and CSK29544_02616-V94A-F and CSK29544_02616-V94A-R for CSK29544_02616 V94A. For the construction of pSK17 expressing mCherry protein, pQE30 harboring *gfp*[3] was digested with BamHI and SacI and ligated with mCherry-encoding sequences PCR-amplified using

mCherry-F-BamHI and mCherry-R-SacI primers. Primers used in this study for plasmids construction are listed in Supplementary Table S3.

Hydrophobicity assay

The hydrophobicity of bacterial surface was measured as described previously.[4] Briefly, bacterial cells were grown overnight, harvested, washed twice with PBS (pH 7.4), and resuspended in 2 ml of PBS. OD_{600} value was measured and recorded as H_0. The bacterial suspension was then mixed with 800 μl of xylene vigorously and incubated at room temperature for 1 h. The OD_{600} value of the aqueous phase was measured and recorded as H. The following formula was used to calculate bacterial hydrophobicity: $[(H_0-H)/H_0] \times 100$.

Autoaggregation (suspension-clearing) assay

Autoaggregation assay was performed according to a previous study.[5] Briefly, bacteria were cultivated overnight, transferred to fresh LB medium at 1%, and incubated at 37° C with constant shaking. Bacteria at mid-log phase of growth were diluted with LB broth to adjust OD_{600} value to 1.5 in a 14 ml of round bottom tube. They were left to sit at room temperature. The rate of suspension clearance was measured by careful removal of 100 μl from the top of the suspension and measurement of its OD_{600} values for 7 h.

LC-MS/MS analysis

A nano LC–MS/MS analysis was performed with a nano HPLC system (Agilent, USA). Nano-chip column (Agilent, USA) was used for peptide separation. Mobile-phase A used for LC separation was 0.1% formic acid in deionized water. Mobile-phase B was 0.1% formic acid in acetonitrile. Chromatography gradient was designed with a linear increase from 3% B to 50% B in 25 min, 90% B in 5 min, and 3% B in 15 min. The flow rate was maintained at 300 nL/min. Product-ion spectra were collected in information-dependent acquisition (IDA) mode. They were analyzed using Agilent 6530 Accurate-Mass Q-TOF with continuous cycles of one full-scan TOF MS from 350 m/z to 1200 m/z (1.0 s) plus two product-ion scans from 100 m/z to 1700 m/z (1 s each). Precursor m/z values were selected starting with the most intense ion using a selection isolation width of ~ 4 Da. Rolling collision-energy feature was used to determine collision energy based on precursor value and charge state. Dynamic exclusion time for precursor-ion m/z values was 20 s.

Database search

Mascot (Matrixscience, USA), an on-line search engine, was used to analyze peptide sequences and identify the proteins. Database search criteria were as follows: taxonomy using *Cronobacter sakazakii* (NCBI nr database downloaded on Nov. 8, 2013), BAA 894 (Accession No.: NC_009778.1), and ATCC 29544 (Accession No.: CP011047.1); fixed modification of "carboxyamidomethylated at cysteine residues";

variable modification of “oxidized at methionine residues”; maximum-allowed missed cleavage of 2; MS tolerance of 100 ppm; and MS/MS tolerance of 0.1 Da. Only trypsin digestion was used for peptide-fragment analysis.

β-galactosidase assay

β-galactosidase assay was performed in duplicates as described elsewhere.[6] Briefly, 1 ml of bacterial culture was centrifuged at 16,000 × g for 1 min and re-suspended in Z-buffer (60 mM Na_2HPO_4, 40 mM NaH_2PO_4, 10 mM KCl, 2 mM $MgSO_4$, and 40 mM β-mercaptoethanol, adjusted to pH 7.0). Its optical density was measured at wavelength of 600 nm. Aliquots (0.1 ml) of the cell suspension were added to Pyrex tubes containing 20 μl of 0.1% SDS, 40 μl of chloroform, and 0.9 ml of Z-buffer, followed by vortex mixing for 10 s. These samples were incubated at room temperature for 10 min with occasional vortex mixing. They were then mixed with 0.2 ml of 2-nitrophenyl β-D-galactopyranoside (ONPG; 4 mg/ml) to develop yellow-colored products. The reaction was quenched by the addition of 0.5 ml of 1 M Na_2HCO_3. These samples were centrifuged at 16,000 × g for 1 min to collect cell debris. The optical density of the supernatant was measured at wavelength of 420 nm or 550 nm. β-galactosidase activity (arbitrary units) was calculated according to conventional method and expressed in Miller units.

Culture conditions for protein purification

Bacterial cultures at OD_{600} of 0.5 to 1 were treated with arabinose and IPTG at final concentrations of 13.3 mM and 0.5 mM, respectively, and incubated for an additional 4 h. For protein purification, cells that produced the proteins of interest were harvested by centrifugation at 10,000 × g, suspended in lysis buffer (20 mM Tris-Cl with 300 mM NaCl, adjusted to pH 8.0) supplemented with protease-inhibitor cocktail (Sigma, Korea), and disrupted by ultra-sonication (Youngjin Corp., Korea). Cellular debris was removed by centrifugation at 21,130 × g for 1 h at 4°C. Soluble crude cytosol fraction was further processed as described below.

Overexpression and purification of His-CSK29544_02616

For nickel chelated nitrilotriacetic acid (Ni-NTA) affinity chromatography (Qiagen, USA), bacterial cells were resuspended in a lysis buffer containing 5 mM imidazole and sonicated on ice. Cell lysates were centrifuged at 21,130 × g for 1 h at 4°C to remove cell debris. The supernatant was subjected to lysis buffer-equilibrated Ni-NTA affinity chromatography. After incubation with gentle end-over-end rotation at 4°C for 1 h, protein-bound Ni-NTA resin was washed 10 times with 1 ml of washing buffer (lysis buffer containing 20 mM imidazole) and twice with 1 ml of washing buffer (lysis buffer containing 50 mM imidazole). His-tagged target proteins were eluted with elution buffer (20 mM Tris-Cl, 300 mM NaCl, and 250 mM imidazole, adjusted to pH 8.0) and

concentrated using Amicon^{R} Ultra-4 (Millipore, USA) according to the manufacturer's instructions. The buffer was changed to storage buffer (20 mM Tris-Cl, 300 mM NaCl, and 50% glycerol, adjusted to pH 8.0) using PD midiTrap^{TM} G-25 (GE Healthcare, UK). Aliquots of protein were then stored at - 20°C until further use.

Purification of LpxA

Free tagged-LpxA was purified as described previously with minor modifications.[7] Briefly, cell extracts in 20 mM potassium phosphate (KPhos) buffer and 20% glycerol (buffer A) were filtered and applied to Green-Separopor E4B-CL (bioWORLD, USA) equilibrated with the same buffer. The column was incubated at 4°C for 18 h using end-over-end rotation. The column was washed three times with 0.5 ml of buffer A and eluted with 0.5 ml of a buffer A containing 0.2 M, 0.3 M, and 1 M NaCl, respectively. Eluted LpxA fractions (1 M NaCl for high purity) were used for further study.

Purification of holo-acyl carrier protein

Holo-ACP was produced in *E. coli* ER2566 cells expressing both apo-ACP and holo-ACP synthetase encoded by *acpP* and *acpS*, respectively, according to a previous study.[8] Holo-ACP was purified in a manner similar to what was described previously with slight modifications.[7] Briefly, cells were cultured at 37°C and induced with 0.5 mM of IPTG at 18°C for 12 h. Bacterial lysates in 10 ml of ACP buffer (20 mM HEPES and 1

mM TCEP (Tris(2-carboxyethyl)phosphine hydrochloride) at pH 8.0) were mixed with 10 ml of 2-propanol (Final concentration of 50%) slowly. They were then incubated at 4°C for 1 h with end-over-end rotation (F1 mode, 12 rpm). The suspension was centrifuged at 13,000 × g for 30 min at 4°C. The resulting supernatant was collected and diluted with 20 ml of ACP buffer. The diluted solution was loaded onto a Source 15Q column (6 ml) and washed with ACP buffer containing gradient NaCl concentrations from 0 mM to 500 mM. SDS-PAGE analysis revealed that holo-ACP was eluted by 300 mM NaCl. The eluent containing holo-ACP was desalted with ACP buffer using PD midiTrap™ G-25 and stored at 4°C. Protein concentration was measured using Bio-Rad protein assay.

Purification of acyl carrier protein synthetase

Bacterial cells were prepared as described above and AasS-His was purified using Ni-NTA resin in a similar way with some modifications as described below. AasS-His eluted from Ni-NTA resin was subsequently desalted using PD midiTrap™ G-25 with a buffer containing 20 mM Tris-HCl (pH 7.5), 10% glycerol, 1 mM ethylenediaminetetraacetic acid (EDTA), 0.1 mM TCEP, and 0.002% Triton X-100 as reported earlier for optimal storage.[9] The desalted-protein solution was aliquoted into micro-centrifuge tubes and stored in a deep-freezer. Protein concentration was measured using Bio-Rad protein assay.

Acylation of holo-ACP

Acylation of holo-ACP was conducted as described previously[9] with slight modifications. Briefly, holo-ACP (40 µM) was first reduced at 25°C for 1 h in the presence of two equivalents of TCEP (80 µM). It was then incubated in acylation-reaction buffer containing 5 mM ATP, 5 mM $MgCl_2$, 100 µM TCEP, 0.01% Triton X-100, 100 µg of AasS, and 150 µM R-3-hydroxymyristic acid in 100 mM Tris (pH 7.5) at 30°C for 50 min. Then it was supplemented with another 50 µg of AasS. After an additional 20 min of incubation, the reactant was loaded onto a Source 15Q column (6 ml) equilibrated with 20 mM HEPES (pH 8.0). The acyl-ACP was eluted at approximately 300 mM NaCl. It was subsequently desalted using the PD midiTrap™ G-25 before its storage at 4°C.

Western-blot analysis

Proteins on SDS-PAGE gels were electro-transferred to PVDF membranes and the membranes were subsequently blocked with TBST buffer (10 mM Tris-Cl, 150 mM NaCl, and 0.1% Tween 20, adjusted to pH 8.0) supplemented with 0.45% skim milk. The blocked membranes were probed with primary anti-GST (3:10,000 dilution; SantaCruz Biotechnology, USA) or anti-His antibodies (3:2,000 dilution; Santa Cruz Biotechnology, USA) for 30 min, and washed three times with 20 ml of TBST buffer. The membranes were treated with secondary antibody solution [TBST buffer supplemented with 0.45%

skim milk and goat anti-mouse IgG-HRP (1:10,000 dilution; Santa Cruz Biotechnology, USA)] for 20 min and then washed with TBST buffer. Chemiluminescence signal was developed using WEST-ZOL plus Western Blot Detection System (iNtRON Biotechnology, Korea) according to the manufacturer's instructions. Signal was then captured using X-ray film exposure.

For analysis of Labp derivatives including W29L and V94A, bacterial cells producing each derivative via induction with 13.3 mM of arabinose for 4 h were ultra-sonicated in a lysis buffer (20 mM Tris-Cl, 300 mM NaCl, and 5 mM imidazole, adjusted to pH 8.0). Lysate aliquots were saved for analysis of whole-cell lysates. The remainders of cell lysates were centrifuged at 21,130 × g for 1 h at 4°C to separate soluble protein fraction from insoluble cellular debris. These protein samples were further subjected to SDS-PAGE and western-blot analyses as described above.

Prediction of Labp structural modeling

The preliminary model of Labp was generated from structure of perosamin N-acetyltransferase (PDB ID: 4EAA)[10] using the structure homology-modeling server SWISS-MODEL workspace.[11] Trimeric structure was manually built from PyMOL and Coot[12] and further refined using GalaxyRefineComplex[13] for better resolution. Final images were generated using UCSF Chimera.[14]

References

1. Datsenko, K. A. & Wanner, B. L. One-step inactivation of chromosomal genes in *Escherichia coli* K-12 using PCR products. *Proc. Natl. Acad. Sci. U S A* **97**, 6640-6645, doi:10.1073/pnas.120163297 (2000).
2. Choi, Y. *et al.* Plasmid-encoded MCP is involved in virulence, motility, and biofilm formation of *Cronobacter sakazakii* ATCC 29544. *Infect. Immun.* **83**, 197-204, doi:10.1128/IAI.02633-14 (2015).
3. Kim, K. P. & Loessner, M. J. *Enterobacter sakazakii* invasion in human intestinal Caco-2 cells requires the host cell cytoskeleton and is enhanced by disruption of tight junction. *Infect. Immun.* **76**, 562-570, doi:10.1128/IAI.00937-07 (2008).
4. Wang, L., Hu, X., Tao, G. & Wang, X. Outer membrane defect and stronger biofilm formation caused by inactivation of a gene encoding for heptosyltransferase I in *Cronobacter sakazakii* ATCC BAA-894. *J. Appl. Microbiol.* **112**, 985-997, doi:10.1111/j.1365-2672.2012.05263.x (2012).
5. Merkx-Jacques, A., Obhi, R. K., Bethune, G. & Creuzenet, C. The *Helicobacter pylori flaA1* and *wbpB* genes control lipopolysaccharide and flagellum synthesis and function. *J. Bacteriol.* **186**, 2253-2265 (2004).

6 Miller, J. H. *Experiments in molecular genetics*. (Cold Spring Harbor Laboratory Press, 1972).

7 Jenkins, R. J. & Dotson, G. D. A continuous fluorescent enzyme assay for early steps of lipid A biosynthesis. *Anal. Biochem.* **425**, 21-27, doi:10.1016/j.ab.2012.02.027 (2012).

8 Broadwater, J. A. & Fox, B. G. Spinach holo-acyl carrier protein: overproduction and phosphopantetheinylation in *Escherichia coli* BL21(DE3), in vitro acylation, and enzymatic desaturation of histidine-tagged isoform I. *Protein Expr. Purif.* **15**, 314-326, doi:10.1006/prep.1998.1016 (1999).

9 Jiang, Y., Chan, C. H. & Cronan, J. E. The soluble acyl-acyl carrier protein synthetase of *Vibrio harveyi* B392 is a member of the medium chain acyl-CoA synthetase family. *Biochemistry* **45**, 10008-10019, doi:10.1021/bi060842w (2006).

10 Thoden, J. B. *et al.* Catalytic mechanism of perosamine N-acetyltransferase revealed by high-resolution X-ray crystallographic studies and kinetic analyses. *Biochemistry* **51**, 3433-3444, doi:10.1021/bi300197h (2012).

11 Biasini, M. *et al.* SWISS-MODEL: modelling protein tertiary and quaternary structure using evolutionary information. *Nucleic Acids Res* **42**, W252-258, d

oi:10.1093/nar/gku340 (2014).

12 Emsley, P., Lohkamp, B., Scott, W. G. & Cowtan, K. Features and development of Coot. *Acta Crystallogr D Biol Crystallogr* **66**, 486-501, doi:10.1107/S0907444910007493 (2010).

13 Heo, L., Lee, H. & Seok, C. GalaxyRefineComplex: Refinement of protein-protein complex model structures driven by interface repacking. *Sci Rep* **6**, 32153, doi:10.1038/srep32153 (2016).

14 Pettersen, E. F. *et al.* UCSF Chimera--a visualization system for exploratory research and analysis. *J Comput Chem* **25**, 1605-1612, doi:10.1002/jcc.20084 (2004).

15 Lee, C. H. & Tsai, C. M. Quantification of bacterial lipopolysaccharides by the purpald assay: measuring formaldehyde generated from 2-keto-3-deoxyoctonate and heptose at the inner core by periodate oxidation. *Anal. Biochem.* **267**, 161-168, doi:10.1006/abio.1998.2961 (1999).

16 Kim, K. *et al.* Outer membrane proteins A (OmpA) and X (OmpX) are essential for basolateral invasion of *Cronobacter sakazakii*. *Appl. Environ. Microbiol.* **76**, 5188-5198, doi:10.1128/AEM.02498-09 (2010).

17 Kim, S. *et al.* Hfq plays important roles in virulence and stress adaptation i

n *Cronobacter sakazakii* ATCC 29544. *Infect. Immun.* **83**, 2089-2098, doi:10.1128/IAI.03161-14 (2015).

18 Hanahan, D. Studies on transformation of *Escherichia coli* with plasmids. *J. Mol. Biol.* **166**, 557-580 (1983).

19 Karimova, G., Pidoux, J., Ullmann, A. & Ladant, D. A bacterial two-hybrid system based on a reconstituted signal transduction pathway. *Proc. Natl. Acad. Sci. U S A* **95**, 5752-5756 (1998).

20 Lee, C. R., Cho, S. H., Yoon, M. J., Peterkofsky, A. & Seok, Y. J. *Escherichia coli* enzyme IIANtr regulates the K+ transporter TrkA. *Proc. Natl. Acad. Sci. U S A* **104**, 4124-4129, doi:10.1073/pnas.0609897104 (2007).

21 Chang, A. C. & Cohen, S. N. Construction and characterization of amplifiable multicopy DNA cloning vehicles derived from the P15A cryptic miniplasmid. *J. Bacteriol.* **134**, 1141-1156 (1978).

22 Guzman, L. M., Belin, D., Carson, M. J. & Beckwith, J. Tight regulation, modulation, and high-level expression by vectors containing the arabinose PBAD promoter. *J. Bacteriol.* **177**, 4121-4130 (1995).

23 Karimova, G., Ullmann, A. & Ladant, D. Protein-protein interaction between *Bacillus stearothermophilus* tyrosyl-tRNA synthetase subdomains revealed by

a bacterial two-hybrid system. *J. Mol. Microbiol. Biotechnol.* **3**, 73-82 (2001).

24 Sheffield, P., Garrard, S. & Derewenda, Z. Overcoming expression and purification problems of RhoGDI using a family of "parallel" expression vectors. *Protein Expr. Purif.* **15**, 34-39, doi:10.1006/prep.1998.1003 (1999).

Figure legends

Figure S1. Attenuated invasion ability of CSK29544_02616::*Tn* and location of transposon insertional site in the clone.

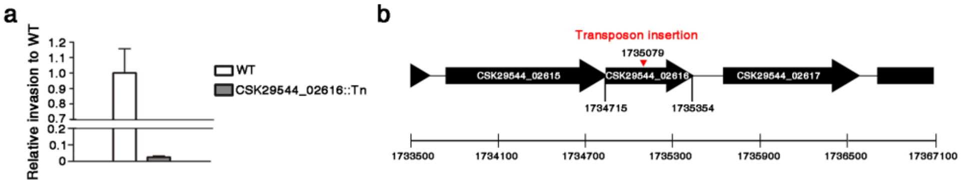

(**a**) Invasion ability of CSK29544_02616::*Tn* was compared with that of WT strain using epithelial Caco-2 cells. The error bars represent standard deviations from three independent biological replicates. (**b**) The location of transposon insertion in the CSK29544_02616::*Tn* clone was identified.

Figure S2. The number of internalized bacteria in RAW264.7 cells co-infected with WT and ΔCSK29544_02616 strains

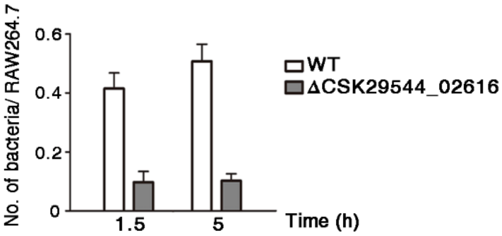

RAW264.7 cells were infected with a 1:1 mix of WT strain expressing green fluorescence protein (GFP) and ΔCSK29544_02616 strain expressing mCherry protein. The number of internalized bacteria per RAW264.7 cell was counted from 20 images of randomly selected regions on cover slips and depicted at indicated time points after infection.

Figure S3. Growth of WT and ΔCSK29544_02616 strains *in vitro*

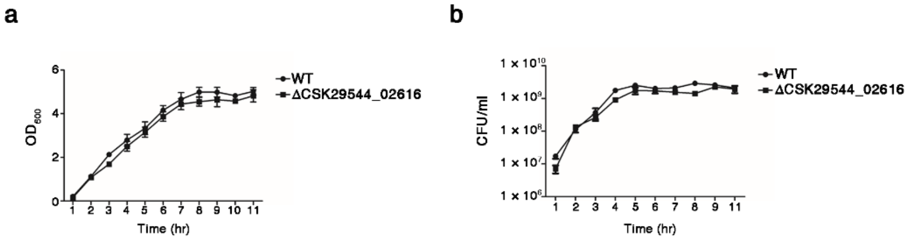

C. sakazakii WT and ΔCSK29544_02616 strains were cultured in LB broth at 37°C. The OD_{600} values (**a**) and viable cell numbers (**b**) of each strain were measured at indicated time points. The error bars represent standard deviations from three independent biological replicates.

Figure S4. Labp conserved among all *Cronobacter* species.

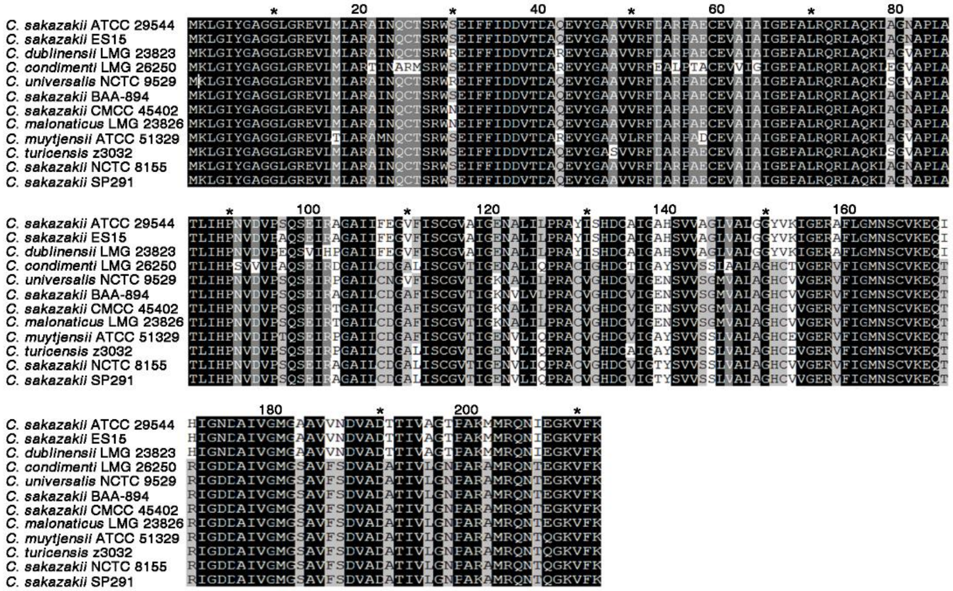

Multiple sequence alignment of Labp and its homologues in all *Cronobacter* species was generated by Clustal Omega (version 1.2.1.) and Genedoc. Amino acids conserved in at least 7 strains are highlighted in grey or black.

Figure S5. Comparison of transcriptional expression of *ompA*, *ompX* and *inv* between WT and ΔCSK29544_02616 strains

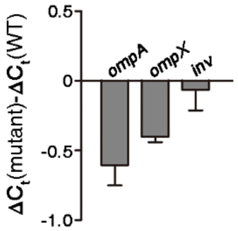

qRT-PCR analysis of invasion-associated genes (*ompA*, *ompX* and *inv*). Transcription levels of each gene were normalized using those of 16S rRNA in each bacterial strain. The normalized expression values were further compared between WT and the mutant strains, and the relative expression values were averaged from three experiments conducted independently.

Figure S6. Quantification of LPS extracted from WT and ΔCSK29544_02616 strains

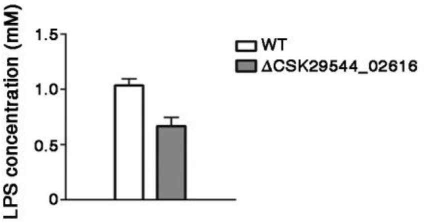

LPS was extracted from WT and ΔCSK29544_02616 strains using hot-phenol-water and quantified using purpald assay.[15] LPS concentrations between strains were normalized using numbers of viable cells used in assays.

Figure S7. Protein purification using affinity chromatography.

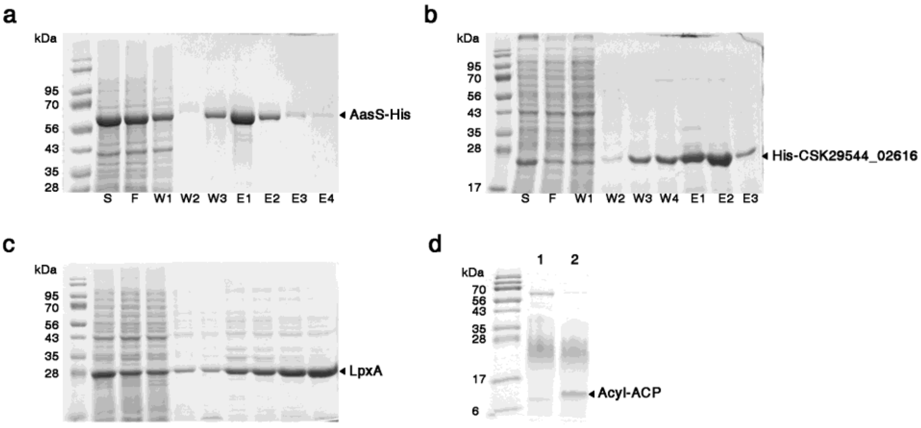

SDS-PAGE analysis was conducted to evaluate the purity of over-expressed proteins. N-terminally $His_{\times 6}$-tagged proteins including AasS (**a**) and CSK29544_02616 (**b**) were purified using Ni-NTA resin while free tagged-LpxA was purified using Green-SeparoporE 4B-CL (**c**). Holo-ACP was bona fide synthesized without a tag in *E. coli* ER2566 producing AcpP (apo-ACP) and AcpS (holo-ACP synthetase). It was purified using anion-exchange chromatography (lane 1 in **d**) and further subjected to AaaS-mediated acylation. The acyl-ACP was purified using anion-

exchange chromatography (lane 2 in **d**).

Figure S8. Optimization of LpxA activity assay

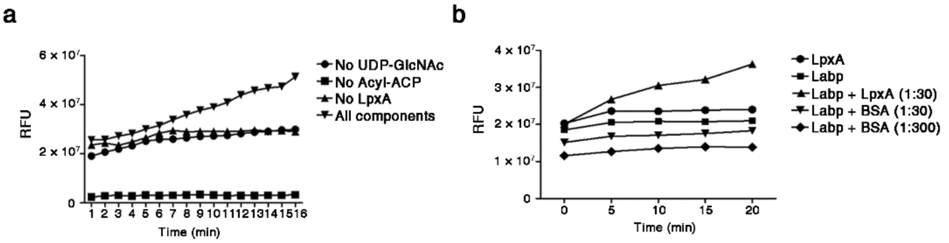

(**a**) LpxA enzyme assay was designed by measuring fluorescence of ThioGlo-ACP conjugates. In an complete reaction containing all components, 20 mM HEPES (pH 8.0), 8 µM *R*-3-hydroxymyristoly-ACP, 4 mM UDP-GlcNAc, 10 µM ThioGlo, and 10 nM LpxA were mixed and incubated at 30°C in a final volume of 100 µl and fluorescent ThioGlo-ACP conjugates were continuously monitored at λ_{ex} = 378 nm and λ_{em} = 446 nm for 2 h at 15 s intervals. Controls included reactions containing all except either UDP-GlcNAc, acyl-ACP, or LpxA. Data are representatives of five independent experiments. (**b**) Reaction-progress curves of the LpxA activity assay in the presence of either Labp or BSA. BSA was used

as a negative control. Ratios in parentheses indicate molar ratio. The assay procedure was equivalent to that stated in (**a**) above.

Figure S9. The predicted model structure of Labp.

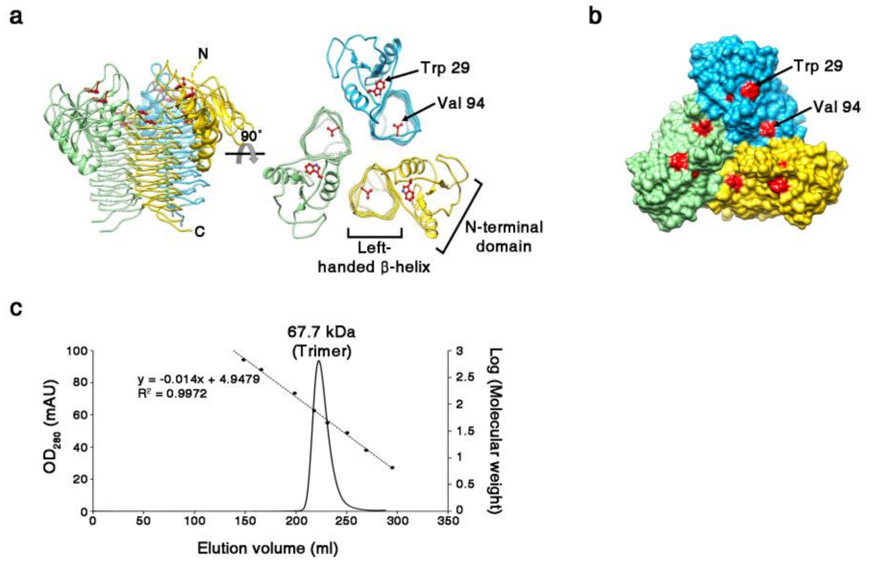

(**a**) Two orthogonal views of the Labp model structure were shown as ribbon diagram (left, side view; right, top view). Each protomer was shown in blue, yellow and green, respectively. The Trp29 and Val94 residues were represented as red ball-and-stick models and indicated with arrows. (**b**) The top surface structure of Labp. The Trp29 and Val94 residues were marked as red color. (a) and (b) models were predicted by

PyMOL and Coot.[11,12] (**c**) Elution profile of the Labp on a size-exclusion chromatography (solid line). The calculated molecule weight of the protein is labeled on the peak, based on the following standard proteins: thyroglobulin (669 kDa), ferritin (440 kDa), aldolase (158 kDa), coalbumin (75 kDa), ovalbumin (44 kDa), carbonic anhydrase (29 kDa), and RNase A (13.7 kDa). The standard curve was shown as dashed line. X-axis indicates elution volume (ml) and Y-axis indicate absorbance at 280 nm (left) and molecular weight on log scale (right).

Figure S10. Full-length images of Figure 3a, 3b and 3c.

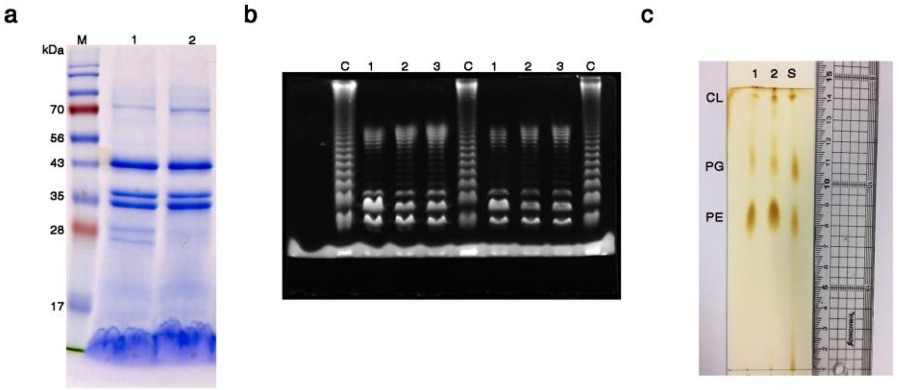

(**a**) Outer membrane protein profiles between WT (lane 1) and ΔCSK29544_02616 (lane 2) strains. Outer membrane fractions equivalent to 10 μg of proteins were analyzed by SDS-PAGE in parallel with molecular-weight (MW) size markers (M). (**b**) Gel image of LPS profiles. Lane 1 indicates WT and Lane 2 and 3 indicate ΔCSK29544_02616 mutant. Lane C shows LPS from *Salmonella* Typhimurium LT2 as a positive control. (**c**) Gel image of PL TLC analysis. Membrane PLs were extracted from equivalent numbers of bacterial cells of WT (lane 1) and CSK29544_02616 (lane 2) strains and separated by TLC analysis. Lane S

indicates purified standard PLs, which were used to identify spots of each PL species.

Figure S11. Full-length images of Figure 5a and 5c.

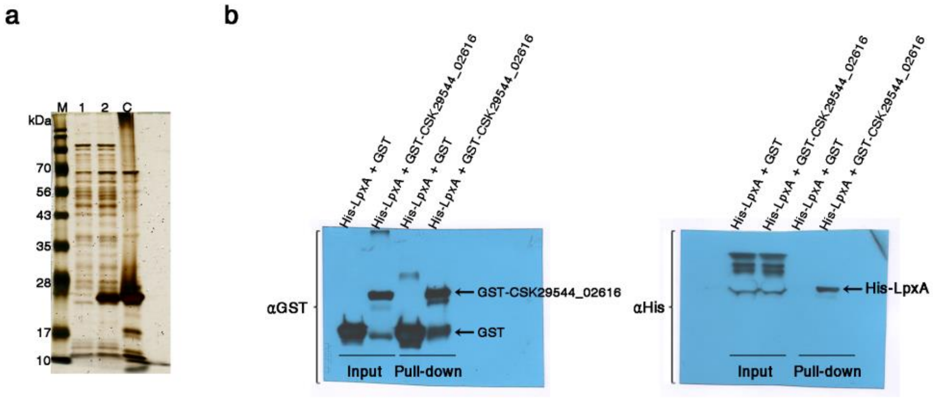

(**a**) Ligand-fishing analysis with His-CSK29544_02616 as bait. Soluble cell extracts from ΔCSK29544_02616 were incubated in the absence (lane 1) or presence of His-CSK29544_02616 (lane 2) and passed through Ni-NTA resin. Eluted proteins along with MW size markers (M) and control (C, purified His-CSK29544_02616) were subjected to 12% SDS-PAGE. (**c**) GST pull-down analysis**.** Total cell lysates were incubated with glutathione (GSH)-agarose beads. Total lysates and GSH pulled-down fractions were subjected to immunoblotting using anti-GST and anti-His

antibodies, respectively.

Figure S12. Full-length image of Figure 7c.

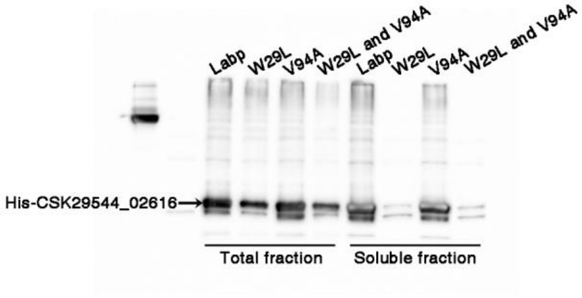

Equivalent amounts of proteins were subjected to SDS-PAGE and transferred to PVDF membranes. The membrane was blotted with anti-His and target proteins were indicated with an arrow.

Table S1. Bacterial strains and plasmids used in this study

Strain or plasmid	Genotype and/or characteristics	Reference or source
C. sakazakii ATCC 29544	Wild-type strain	[16]
ES1001	29544 harboring pKD46	[16]
SK001	29544 harboring pACYC184	[17]
SK009	29544 harboring pBAD24	This study
SK010	CSK29544_02616::*kan*	This study
SK011	ΔCSK29544_02616	This study
SK012	ΔCSK29544_02616 harboring pACYC184	This study
SK013	ΔCSK29544_02616 harboring pSK01	This study
SK014	ΔCSK29544_02616 harboring pBAD24	This study
SK015	ΔCSK29544_02616 harboring pSK02	This study
SK016	29544 harboring pQE30::*gfp*	This study
SK017	ΔCSK29544_02616 harboring pSK16	This study
E. coli		

DH5α	λ^- Φ80d*lacZ*Δ(*lacZYA-argF*)*U169* *recA1 endA1 hsdR17*(r_K^- m_K^-) *supE44* *thi-1 gyrA relA1*	[18]
BTH101	F^- *cya-99 araD139 galE15 galK16* *rpsL1*(Str^R) *hsdR2 mcrA1 mcrB1*; reporter strain in bacterial two-hybrid assay	[19]
BL21 (DE3)	F^- *ompT hsdS* (r_B^- m_B^-) *gal* (DE3); protein overexpression	Laboratory collection
ER2566	F^- λ^- *fhuA2 [lon] ompT lacZ::t7 gene1* *gal sulA11* Δ*(mcrC-mrr)114::IS10* *R(mcr-73::miniTn10-TetS)2 R(zgb-210::Tn10--TetS) endA1 [dcm]*	[20]
SK016	BTH101 harboring pKT25 and pSK04	This study
SK017	BTH101 harboring pSK03 and pSK04	This study
SK018	BL21(DE3) harboring pSK06	This study
SK019	ER2566 harboring pSK05	This study

SK020	ER2566 harboring pSK08	This study
SK021	ER2566 harboring pSK07	This study
SK022	BL21(DE3) harboring pSK09	This study
SK023	BTH101 harboring pSK04 and pSK10	This study
SK023	BTH101 harboring pSK04 and pSK11	This study
SK024	BTH101 harboring pSK04 and pSK15	This study
SK025	DH5α harboring pSK12	This study
SK026	DH5α harboring pSK13	This study
SK027	DH5α harboring pSK14	This study
SK028	BTH101 harboring pSK03 and pSK15	This study
**Plasmids**		
pKD13	*oriR6K* Amp^R FRT Kan^R FRT	[1]
pKD46	*oriR101 repA101*(Ts) Amp^R *ara* *BADpgam-bet-exo*	[1]
pCP20	*ori*pSC101(TS) Amp^{R}Cm^R cI857λ $P_R$flp	[1]
pACYC184	Tet^R Cm^R p15A *ori*	[21]
pBAD24	Amp^R, *araC*, P_{BAD}, pBR322 *ori*,	[22]

	expression vector	
pKT25	*ori* p15A, *Plac*::*cyaA* 1–224, Kan^R	[23]
pUT18C	ColEI-ori, *Plac*::*cyaA* 225–399, MCS, Amp^R	[23]
pKT25-zip	*ori* p15A, *Plac*::*cyaA* 1–224ΦGCN4-zip, Kan^R	[23]
pUT18C-zip	ColEI-ori, *Plac*::*cyaA* 225–399ΦGCN4-zip, Amp^R	[23]
pETDuet 1	LacI, pBR322ori, pT7, two MCS, Amp^R, expression vector	Novagen
pGST parallel 1	LacI, tac promoter, $Amp^{R,}$, expression vector	[24]
pSK01	pACYC184-CSK29544_02616	This study
pSK02	pBAD24- His-CSK29544_02616	This study
pSK03	pKT25-CSK29544_02616	This study
pSK04	pUT18C-*lpxA*	This study
pSK05	pETDuet 1-His-*lpxA*	This study

pSK06	pGST parallel 1-GST-CSK29544_02616	This study
pSK07	pETDuet 1- AcpP and AcpS	This study
pSK08	pETDuet 1-*lpxA*	This study
pSK09	pET28a-*aasS*-His	This study
pSK10	pKT25-CSK29544_02616 (W29L)	This study
pSK11	pKT25-CSK29544_02616 (V94A)	This study
pSK12	pBAD24- His-CSK29544_02616 (W29L)	This study
pSK13	pBAD24- His-CSK29544_02616 (V94A)	This study
pSK14	pBAD24- His-CSK29544_02616 (W29L, V94A)	This study
pSK15	pUT18C-*lpxD*	This study
pSK16	pQE30::*gfp*	[3]
pSK17	pQE30::mCherry	This study

Table S2. Proteins identified in ligand fishing

Gene	Locus tag	Function	Molecular weight (kDa)
nanK	CSK29544_00587	N-acetylmannosamine kinase	29
rpsC	CSK29544_01296	30S ribosomal protein S3	26
trmB	CSK29544_01586	tRNA (guanine-N(7)-)-methyltransferase	27
nudC	CSK29544_00647	NADH pyrophosphatase	30
folB2	CSK29544_03238	Dihydromonapterin reductase	27
	CSK29544_03652	Putative hydrolase	27
labp	CSK29544_02616	LpxA-binding protein	22

Table S3. Primers used to construct bacterial strains and plasmids in this study

Oligonucleotide name	Oligonucleotide sequences (5' to 3')
CSK29544_02616-lamb-F	CGC GTG GTG GAT GCT ATC CGC GCG CAG GCC GCG CTC GGC CTT GCG GAG AAA GTG GCA TGA TGT AGG CTG GAG CTG CTT CG
CSK29544_02616-lamb-R	CGT CAT TGA CTA CTG CCG CTC CCA TGC CAA CGA TAG CGT CGT TAC CGA TGT GGA TTT GCT ATT CCG GGG ATC CGT CGA CC
His- CSK29544_02616-F	AAA GAA TTC ATG CAT CAT CAT CAT CAT CAC GGC AGC GGC AGC GGC AGC GGC AGC ATG AAG CTT GGC ATT TAC GGC G
His- CSK29544_02616-R	AAA GTC GAC CAT CTT CAA TAA CAG ACA CCG CA
pBAD24-seq-F	GGA TCC TAC CTG ACG CTT TT
pBAD24-seq-R	TTA TCA GAC CGC TTC TGC GT
CSK29544_02616-F-SphI	AAA GCA_TGC GGG AAA CGA CCG TTG TGG C
CSK29544_02616-R-BamHI	AAA GGA TCC CCG GCA CTT CAA AGG TTT CC

pACYC184-seq-F	CTA CTT GGA GCC ACT ATC GAC T
pACYC184-seq-R	TGT CCT ACG AGT TGC ATG ATA
CSK29544_02616-F-BamHI	AAA GGA TCC TGG CAT GAA GCT TGG CAT TT
CSK29544_02616-R-EcoRI	ATA GAA TTC CCA TCT TCA ATA ACA GAC ACC
pKT25-con-F	GCC ATT ATG CCG CAT CTG
pKT25-con-R	CTT CGC TAT TAC GCC AGC
lpxA-F-SalI	AAA GTC GAC CGT GAT TGA TAA GAC CGC CT
lpxA-R-BamHI	AAA GGA TCC CGA CAA AAC GCG CGT TCG
pUT18C-con-F	CGT TCG AAG TTC TCG CCG
pUT18C-con-R	CTG GCT TAA CTA TGC GGC AT
pGST parallel 1-con-F	CCA GCA AGT ATA TAG CAT GG
pGST parallel 1-con-R	CAG GCT CTA GAT TCG AAA G
lpxA-F-BamHI	GAG GCC GGA TCC_AGT GAT TGA
lpxA-R-HindIII	CGT TCG GCA AGC TTG CTT T
acpS-F-NcoI	AAA CCA TGG CGA TTC TGG GGC TCG GTA CC
acpS-R-EcoRI	GAT CCC CAA CAC GAA TTC TAA GGA
acpP-F-NdeI	AAA CAT ATG AGC ACT ATC GAA GAA

acpP-R-XhoI	AAA CTC GAG GTA GAT ACT TGT GGG ACT AAA A
lpxA-F-NdeI	AAA CAT ATG ATT GAT AAG ACC GCC TTT
lpxA-R-XhoI	AAA CTC GAG GAC CGG CAC CAA GAA TAT C
pETDuet1-con-F	TCT CGA TCC CGC GAA ATT AA
pETDuet1-con-R	GGC CGT GTA CAA TAC GAT TA
aasS-F-NcoI	TTT CCA TGG ATA TGA ACC AGT ATG TAA ATG A
aasS-R-XhoI	TTT CTC GAG CAG ATG AAG TTT ACG CAG TTC
CSK29544_02616-W29L-F	GCT TGA GCG AGA TTT TCT TTA TTG AC
CSK29544_02616-W29L-R	GTC AAT AAA GAA AAT CTC GCT CAA GC
CSK29544_02616-V94A-F	CGA ATG TCG ATG CCC CGT CGC AAA GC
CSK29544_02616-V94A-R	GCT TTG CGA CGG GGC ATC GAC ATT CG
mCherry-F-BamHI	AAA GGA TCC ATG GTG AGC AAG GGC GAG
mCherry-R-SacI	AAA GAG CTC TTA CTT GTA CAG CTC GTC CAT G
